# Lipidomic profiling of Arabidopsis chloroplast protein phosphatase SLP1 mutants reveals altered diurnal lipid remodeling

**DOI:** 10.1016/j.bbadva.2026.100180

**Published:** 2026-01-09

**Authors:** Chris White-Gloria, Greg B. Moorhead, Adriana Zardini Buzatto

**Affiliations:** a Department of Biological Sciences, University of Calgary, Calgary, T2N 1N4, Canada; b Calgary Metabolomics Research Facility (CMRF), University of Calgary, Calgary, T2N 1N4, Canada

**Keywords:** Arabidopsis thaliana, Lipid profiling, Chloroplast, Protein phosphatase, Shewanella-like protein phosphatase 1, Lipidomics

## Abstract

Lipids are fundamental to the biology of all organisms, including the functioning of subcellular organelles. The plant chloroplast is the site of photosynthesis and serves as a hub of metabolic events that are dynamically regulated between the light and dark periods. The regulation of chloroplast events, like most other biological processes, is often controlled by protein phosphorylation. Here, we explored the lipidome of *Arabidopsis thaliana* under both light and dark conditions, examining lipid changes upon overexpression or knockout of the chloroplast-specific phosphatase *Shewanella*-like protein phosphatase 1 (SLP1). To date, no substrates have been identified for the phosphatase SLP1. Results indicate that *Arabidopsis thaliana* possesses a dynamic lipidome that undergoes significant changes between light and dark conditions, and that protein phosphorylation, in part controlled by SLP1, plays a crucial role in regulating chloroplast metabolism and its associated lipidome. Alterations due to loss or over-expression of SLP1 in the chloroplast are transmitted to other cellular compartments, leading to altered lipids throughout the cell. We report that although both SLP1 mutant lines display perturbed glycerolipid metabolism, knockout and overexpression affect distinct pathways. In the three *Arabidopsis* lines studied, we also identify dramatic diurnal changes in the abundance of oxidized lipids. Together, these findings establish SLP1-dependent dephosphorylation as an upstream regulator of diel lipid remodelling, linking chloroplast activity to broader cellular lipid organization.

## Highlights

• Reversible protein phosphorylation is a key regulatory mechanism controlling most aspects of cell biology.
• Arabidopsis thaliana possesses a dynamic lipidome that undergoes significant changes between light and dark conditions.
• Chloroplast Shewanella-like protein phosphatase 1 (SLP1) plays a crucial role in regulating the metabolism of the chloroplast and its associated lipidome.
• Alterations due to loss or over-expression of SLP1 in the chloroplast are transmitted to other cellular compartments, leading to altered lipids throughout the cell.

## 1 Introduction

Lipids are essential to photosynthetic organisms, functioning as an energy reserve, producing signalling molecules, and forming membranes that segregate the cell into compartments [1]. In photosynthetic tissues, chloroplasts are metabolically specialized organelles that produce and maintain their own distinct lipid repertoire [2]. Thylakoid membranes, which house the photosynthetic machinery, are uniquely composed of the non-phosphorus glycolipids monogalactosyldiacylglycerol (MGDG) and digalactosyldiacylglycerol (DGDG). These galactolipids are synthesized within the chloroplast envelope and are essential for the stability and function of photosystem complexes [3,4].

The chloroplast is the site of *de novo* fatty acid synthesis in plants, producing a substantial fraction of precursors required for extraplastidic lipids, including phospholipids and acylglycerols. Chloroplasts also house the methylerythritol phosphate (MEP) pathway for isoprenoid biosynthesis, producing precursors for prenol lipids, chlorophylls, carotenoids, and other specialized metabolites [5,6]. In contrast, the bulk of phospholipid biosynthesis occurs at the endoplasmic reticulum (ER), requiring a coordinated flux of intermediates between the chloroplast and the ER [7]. This compartmental interdependence supports developmental and stress respoenses but also creates substantial biochemical complexity.

*Arabidopsis* leaf lipidomes have been characterized in depth, with galactolipids dominating the chloroplast glycerolipid fraction and many plastid-derived species carrying highly unsaturated C18 acyl chains. Plant lipidomes are chemically diverse, containing oxidized fatty acids, unusual galactolipids, and prenol-derived metabolites that are often absent from reference databases [8]. Overlapping molecular species synthesized in different organelles and strong diurnal shifts in lipid metabolism further complicate annotation and quantification. These factors make accurate quantification and interpretation of plant lipidomes more difficult than in mammalian or microbial systems [9].

Remodelling of lipid composition is driven, in part, by light-dark cycles, temperature, developmental stage, and stress [[10], [11], [12], [13], [14], [15]]. These conditions alter headgroup distribution, fatty acid saturation, and oxidation states across membranes. Transcriptional regulation contributes to these shifts, but many enzymes involved in lipid synthesis and modification are regulated post-translationally [16,17]. Protein phosphorylation is one such mechanism, but its role in plastid lipid metabolism remains poorly understood. Although several protein kinases have been localized to the chloroplast, most lack functional assignments or known substrates [[18], [19], [20], [21]]. The thylakoid-associated protein kinase STN7, for example, is known to regulate light-state transitions [22], but comparable regulatory pathways for lipid synthesis remain speculative [23]. Chloroplast-localized protein phosphatases are even less understood, representing a major gap in our knowledge of plastid biochemistry.

Recently, we identified *Shewanella-*like protein phosphatase 1 (SLP1), a member of the PPP family, as a chloroplast-specific protein encoded in the nuclear genome [24,25]. We hypothesized that SLP1 influences lipid metabolism within the chloroplast and may indirectly affect extraplastidic lipid pools through altered metabolite flux or signalling.

Previous lipidomics studies have compared plant lipidomes from light and dark, revealing substantial diel shifts [[26], [27], [28]]. Building on this, we used untargeted lipidomics to profile *Arabidopsis thaliana* rosettes from SLP1 knockout and over-expressor lines harvested in matched light and dark periods. Untargeted analysis provides the broad coverage required for discovery-driven mapping of lipid networks and enables the detection of unexpected or low-abundance species that would be missed by predefined targeted panels. Our goal is not to reconstruct the complete *Arabidopsis* lipidome or define direct biochemical targets of SLP1, but rather to test the broader hypothesis that chloroplast protein phosphorylation contributes to diurnal lipid remodeling and inter-organelle metabolic coordination. This approach enabled us to investigate whether SLP1 contributes to the regulation of lipid metabolism under physiological light-dark cycling and to assess the extent to which a chloroplast-localized protein phosphatase can influence lipid pools throughout the cell.

## 2 Materials and methods

### 2.1 Plant materials

**Fig. 1:**
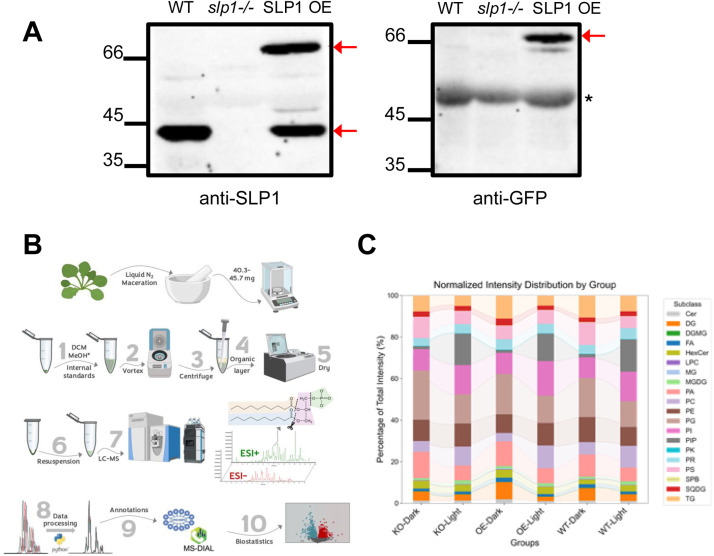
Overview of untargeted lipidomics to study the influence of chloroplastic protein phosphatase *Shewanella*-like phosphatase 1 (SLP1) on the lipidome of *Arabidopsis* rosettes. A) Western blotting confirms the presence or absence of endogenous (left) or RFP-tagged SLP1 (right) in WT, SLP1 knockout (*slp1-/-*), and over-expressor (SLP1 OE) lines. The starred (*) band represents non-specific binding to an abundant protein. B) Experimental workflow: rosette tissue from each plant line was harvested 6 h into the light or dark. Extracted lipids were then subject to analysis by LC-MS/MS and downstream biostatistics. C) The relative summed intensities of all lipids within each class were calculated for each group (i.e., percentage contribution of each lipid class to total abundance). Data are shown for wild-type (WT), SLP1 knockout (KO), and SLP1 over-expression (OE) lines under light and dark conditions.

Numerous T-DNA exon insertional mutant lines were obtained for SLP1 (At1g07010) and screened by Western blot using a SALK knockout line. The SLP1 KO line (*slp1-/-*) and over-expressor SLP1 plants (SLP1 OE; previously described in Uhrig et al. [25]) were confirmed in two consecutive generations using affinity-purified SLP1 antibodies. *Arabidopsis thaliana* ecotype Columbia (Col-0) wildtype (WT), the SLP1 over-expressor line containing SLP1-RFP, and SLP1 KO line were grown in a growth chamber with 16 h of light (21 °C) and 8 h of dark (19 °C). Six hours into the light or dark cycle, rosette leaves were harvested, flash-frozen in liquid nitrogen, and stored at −80 °C. Prior to lipid analysis, a crude extract of each line was generated by grinding the rosettes in 100 μL of SDS-PAGE sample buffer using a micropestle. The extract was then heated at 98 °C for 10 min, followed by centrifugation at 6000 rpm for 10 min. Samples (15 µL) were run on 10 % SDS-PAGE and blotted to nitrocellulose to confirm loss of or over-expression of SLP1 (Fig. 1A). One blot was probed with affinity-purified anti-SLP1 antibody [25] and the other with anti-GFP (GenScript) using the manufacturer’s instructions.

### 2.2 Lipid extractions

*Arabidopsis thaliana* samples (*n* = 7 per group: WT, *slp1-/-* (KO), and SLP1 OE) were grown under controlled conditions and harvested as described. The tissue was flash-frozen and immediately macerated in liquid nitrogen. The tissue was maintained under cryogenic conditions during pulverization, and the lipid extraction solvent was added immediately after weighing. No deliberate thaw step occurred prior to immersion in methanol and dichloromethane. All manipulations of dark cycle samples were conducted in the absence of light, using green light in a dark room. Samples were randomized for preparation and analysis to minimize batch effects.

Each homogenate was extracted in duplicate using a modified Folch protocol adapted to small tissue amounts (40.3–45.7 mg). The classical Folch method employs a chloroform/methanol (2:1, v/v) mixture, followed by water, resulting in a final solvent ratio of 8:4:3 chloroform/methanol/water [29,30]. This solvent system extracts a wide range of lipid classes but requires adjustments of the sample/solvent ratio for limited biomass [[31], [32], [33], [34], [35]]. Chloroform was substituted with dichloromethane due to its lower toxicity and regulatory burden [36,37]. Dichloromethane offers comparable lipophilicity, promoting efficient phase separation. Methanol disrupts lipid-protein interactions and enhances the solubility of amphiphiles. The 0.2 M KCl aqueous phase removes polar contaminants and causes amphiphilic lipids to shift into the organic phase through salting-out.

Tissue aliquots (40.3–45.7 mg) were mixed sequentially with internal standards (0.2 µL/mg; Avanti SPLASH Lipidomix mass spec standard), methanol (6.5 µL/mg), dichloromethane (13.3 µL/mg), and 0.2 M KCl (4.0 µL/mg). Each addition was followed by vortexing for 30 s. Samples were equilibrated at 4 °C for 10 min, then centrifuged at 12,000 rpm (4 °C, 10 min). An aliquot of the organic phases was collected, evaporated to dryness using a SpeedVac at 4 °C, and stored at –80 °C, protected from light, until analysis. The remaining organic layers were pooled and split into equal aliquots for quality control (QC).

The extracts were resuspended in chromatographic mobile phases at a ratio of 35 % mobile phase B and 65 % mobile phase A, resulting in a 20-fold dilution of the original sample (mg of plant tissue / µL of solvent). Extracts were kept at 4 °C in 250 µL polypropylene inserts inside 2 mL amber vials, sealed with PTFE/silicone septa, and injected within 4–36 h post-extraction. We acknowledge that the use of plasticware with organic solvents is not ideal, although it is more practical and cost-effective than glassware [31]. To minimize potential contamination, all plastic components originated from the same production lot and were pre-screened for background contamination [32]. Blank extractions were used to monitor solvent and plastic-derived signals.

Internal standards (Avanti SPLASH Lipidomix, a mixture of 14 deuterated lipids from different classes, including [D7]LPC 18:1, [D7]LPE 18:1, [D7]MG 18:1, [D7]ST 27:1;O (cholesterol-d7), [D7]CE 18:1, [D7]PI 15:0_18:1, [D7]PS 15:0_18:1, [D7]PG 15:0_18:1, [D7]PA 15:0_18:1, [D7]PC 15:0_18:1, [D7]PE 15:0_18:1, [D7]DG 15:0_18:1, [D7]TG 15:0_18:1_15:0, and [D9]SM 18:0;O2/18:1) were used to assess mass detection accuracy, experimental reproducibility, and retention time variation. These standards were added to all samples before extraction to correct for ion suppression, matrix effects, extraction variability, and instrument drift. Hence, they were used exclusively for quantitative normalization and correction of technical variations. Lipid annotations were performed independently and were not influenced by internal standards.

The 42 samples (7 per group: knockout – KO dark, KO light, overexpression – OE dark, OE light, wild-type – WT dark, and WT light) were randomly split into four batches of 10 or 11 for extraction and injection. One replicate of a QC pool, composed of organic extracts from all samples, was resuspended and injected with each sample batch. Extra QC aliquots were injected before, between, and after the sample set, for a total of seven QC technical replicates. The QC injections had the same biological composition; hence, they were used for batch correction and to assess the technical reproducibility of the dataset through relative standard deviations (RSD; i.e., features with RSD exceeding 30 % for QCs were excluded) and clustering through Principal Component Analysis (PCA) and dendrograms.

### 2.3 LC-MS analysis for untargeted lipidomics

Lipid extracts were analyzed by reversed-phase liquid chromatography using a Thermo Vanquish Horizon UHPLC with a Waters Acquity Premier CSH C18 column (1.7 µm, 2.1 × 100 mm), coupled to a Thermo Q-Exactive HF Hybrid Quadrupole-Orbitrap mass spectrometer. Each sample was injected in both positive and negative ionization modes. Injection volumes were 5 µL (positive) and 10 µL (negative). Gradient separation was carried out over 16.5 min, increasing MPB from 10 % to 99 %. Flow rates ranged from 0.270 to 0.350 mL/min. The column temperature was maintained at 47 °C . Full-scan MS was acquired from *m/z* 140 to 2000 at a resolution of 120,000. Data-dependent MS/MS was acquired at a resolution of 45,000. The raw data have been deposited in the MetaboLights repository with the study identifier MTBLS13371 [38].

Mobile phase A (MPA) was composed of 10 mM ammonium formate in methanol/acetonitrile/water (2:2:1), while mobile phase B (MPB) was 10 mM ammonium formate in 2-propanol/acetonitrile/water (96:3:2). Due to the hydrophobic nature of most lipids, strong organic solvents are required for efficient elution. The combination of methanol, acetonitrile, and water in MPA provides enhanced selectivity via hydrogen bonding and π-interactions, while 2-propanol in MPB ensures solubility and elution of hydrophobic lipids. Because many lipids ionize poorly under soft ionization, they are typically detected as adducts. Ammonium formate was added to both mobile phases to enhance adduct formation and improve detection across lipid classes.

Chromatograms were processed using in-house software (Buzatto Research Group, University of Calgary), performing retention time correction, peak picking, alignment, polarity merging, annotation, and normalization. The internal standards added to each sample before extraction served as stable reference points to assist the software’s retention-time correction algorithm during peak alignment. Features present in fewer than 85 % of samples in at least one group were excluded. Known contaminants, adduct redundancies, and low-intensity noise were removed. When lipids were detected in both ionization modes, the mode with the higher peak height was retained.

Lipid features were putatively annotated using established databases, including MS-DIAL LipidBlast, and LIPID MAPS [[39], [40], [41]]. Lipid names conformed to LIPID MAPS and guidelines from the International Lipidomics Society [[42], [43], [44]]. Internal standards were not used for MS/MS spectral matching or structural identification; high-confidence identifications were based solely on experimental MS/MS spectra matched to well-established databases. High-confidence annotations required MS/MS spectral scores ≥400 (0–1000 scale) and mass-to-charge (*m/z*) errors within 3.0 ppm or 2.5 mDa for precursors. Features without MS/MS were annotated based on accurate mass (±3.0 ppm or 2.5 mDa) (low-confidence annotations). It is worth noting that lipid annotations based on mass matches may result in misassignments due to isomeric/isobaric overlaps and are considered tentative. All annotations were filtered using expected retention time windows, ionization patterns, and lipid class–specific criteria, including the biological context (i.e., the expected lipid composition of each sample type) [44]. For each lipid class, annotation was restricted to features within empirical retention-time windows defined by a set of lipid standards. These RT windows were originally optimized on non-plant lipids and later applied here. Consequently, some bona fide plant lipids, particularly highly unsaturated galactolipids and plant sphingolipid species, eluting outside these windows were not annotated and remain in the “unknown feature” pool. A list of annotated features prior to retention time windows is available in Supplementary File S1.

The aligned results were batch-corrected using the QC replicates (pool or organic extract from all samples). Annotated lipids were matched to the most similar internal standard by structural similarity. For example, all lipids annotated as phosphatidylcholines (PC) were normalized by the deuterated standard [D7]PC 15:0_18:1, whereas all lipids annotated as phosphatidylethanolamines were normalized by [D7]PE 15:0_18:1. When an exact class match was not possible, the most similar standard was employed (e.g., a [D7]DG 15:0_18:1 standard was used for normalizing MGDG lipids). Peak ratios (peak height divided by the intensity of the matched standard) were calculated and filtered for QC replicates with relative standard deviation (RSD) <30 %. The internal standard-corrected data were further normalized to the median within each sample (Supplementary File S2 – Table 1). Unidentified compounds were not used for statistical analysis (Supplementary File S2 – Table 2).

All quantitative values reported in this study represent internal-standard- and median-normalized intensities for relative quantification (Supplementary File S2 – Table 1), not absolute abundances. The numerical range of reported lipid normalized intensities is substantially narrower than that of the underlying raw peak areas. Internal-standard normalization corrects for extraction variation, ionization efficiency, ion suppression, and drift, while median normalization minimizes between-sample scaling differences. These steps produce values suitable for statistical comparison but do not preserve absolute lipid-pool proportions or the full raw-intensity span. Therefore, the normalized values cannot be interpreted as absolute abundance ratios between lipid species or as mol % compositions.

### 2.4 Statistics

The internal standard and median-normalized peak ratios for annotated lipids (Supplementary File S2 – Table 1) were auto-scaled and tested for normality (Shapiro-Wilk) and homogenous variances (Levene’s test). Lipidomes were visualized and compared using univariate methods (heatmaps, *t*-tests, volcano plots, analysis of variance - ANOVA), multivariate models (principal component analysis - PCA, and partial least squares discriminant analysis - PLS-DA), and supervised machine learning (Random Forest) for exploratory pattern recognition and classification. Significance thresholds were defined as fold-change (FC) ≥1.50 or ≤0.667, raw p-value < 0.05, and FDR-adjusted p-value (Benjamini–Hochberg correction) < 0.10 [45]. Because SLP1 is a regulatory phosphatase rather than a biosynthetic enzyme, the expected effect sizes are modest. We therefore selected statistical thresholds appropriate for detecting small but reproducible shifts at lipid molecular or species level, which are mechanistically more informative than bulk-class-wide changes. These thresholds are aligned with those routinely used in large-scale LC–MS lipidomics studies [32]. Significant changes are internally coherent across lipid pathways and consistent across replicates, supporting their biological relevance. Heatmaps were constructed with the top lipids selected by one-way ANOVA or Random Forest.

## 3 Results

### 3.1 Workflow, SLP1 knockout and over-expressor lines

SLP1 is highly expressed in *Arabidopsis* photosynthetic tissues (e.g., rosettes) but not in non-photosynthetic tissues (e.g., roots) [25], making rosettes the focus of our study. Prior to lipidomics, a crude rosette extract was generated from each line. Samples were run on SDS-PAGE, then blotted with affinity-purified anti-SLP1 or GFP antibody (which also recognizes RFP) to ensure SLP1 knockout (KO or *slp1-/-*) and over-expression (SLP1 OE) (Fig. 1A). Endogenous SLP1 runs at ∼42 kDa, while the over-expressed SLP1-RFP is ∼67 kDa [25]. We have previously shown that the C-terminal RFP tagged SLP1 resides in the chloroplast as expected [25]. After confirming the expected SLP1 expression, *Arabidopsis* rosettes were harvested at 6 h light and dark time points in a 16/8 diel cycle, followed by lipid extraction and LC-MS/MS analysis (Fig. 1B).

### 3.2 Lipid data annotation and principal component analysis (PCA) of WT and SLP1 mutant lipidomes

Lipidomics of *Arabidopsis thaliana* tissue resulted in the annotation of 418 lipids (Supplemental Fig. S1**,** Supplementary File S2 **–** Table 1). These include well-resolved species from major membrane and signalling lipid classes, such as galactolipids (MGDG, DGDG), phospholipids (PC, PE, PI, PG, PS, PA), prenol lipids (PR), free fatty acids (FA), triacylglycerols (TG), and oxidized derivatives. Of these, 117 met our criteria for high-confidence annotation, defined as MS/MS spectral similarity scores ≥400 with *m/z* error ≤2.5 ppm. Supplementary File S2 provides a list of annotated (Table 1) and unknown features (Table 2) with normalized intensities, and Supplementary File S1 reports the raw (non-normalized) peak intensities for all detected features (unannotated or annotated without retention time filtering).

As expected for photosynthetic tissues, galactolipids and phospholipids dominated the total raw signal (Supplementary File S1, Supplemental Fig. S2). Arabidopsides A, B, D, and G (oxidized galactolipids derived from chloroplast MGDG and DGDG) were among the most intense species in the raw annotated dataset. Across wild-type plants, MGDG lipids accounted for 24.1 % of all raw annotated lipid intensities and 34.3 % of the polar lipidome 6 h into the light period. In the dark, they remained the dominant class (21.4 % of total raw intensities and 30.5 % of polar lipids). However, these proportions reflect raw signal distributions among all annotated features and are not absolute mol % lipid compositions. Normalization is essential for LC-MS comparative analysis because detector response is nonlinear, and raw intensities cannot be directly compared across runs or classes. Furthermore, the class-level distributions discussed in this work use normalized intensities for annotated features that passed retention-time filters. Several high-abundance *Arabidopsis* galactolipids (for example, MGDG 34:6 and 36:6) are clearly detectable in the raw data (Supplementary File S1) but eluted outside the retention time windows used for annotation. These species are therefore present in the raw dataset but are under-represented in the normalized annotated class totals, consistent with the limitations of untargeted workflows and the need for retention time-based filtering to avoid misassignment.

To assess broad trends in lipid distribution, we summed the normalized intensities across lipid classes for each condition and genotype after filtering (Fig. 1C), revealing differences between light and dark conditions, as well as between *slp1*-/- and SLP1 OE. MGDG and DGDG levels remained relatively stable across conditions, suggesting limited light–dark regulation of the bulk thylakoid lipid pool, although some molecular species were significantly altered for the dark/light comparison (e.g., MGDG 16:0_18:3, MGDG 16:1_16:3). In contrast, phosphatidic acid (PA), phosphatidylcholine (PC), and phosphatidylinositol (PI) showed strong diurnal regulation and differed between WT and mutant lines. Knockout and over-expression lines both displayed distinct disruptions in the balance of phospholipid classes.

**Fig. 2:**
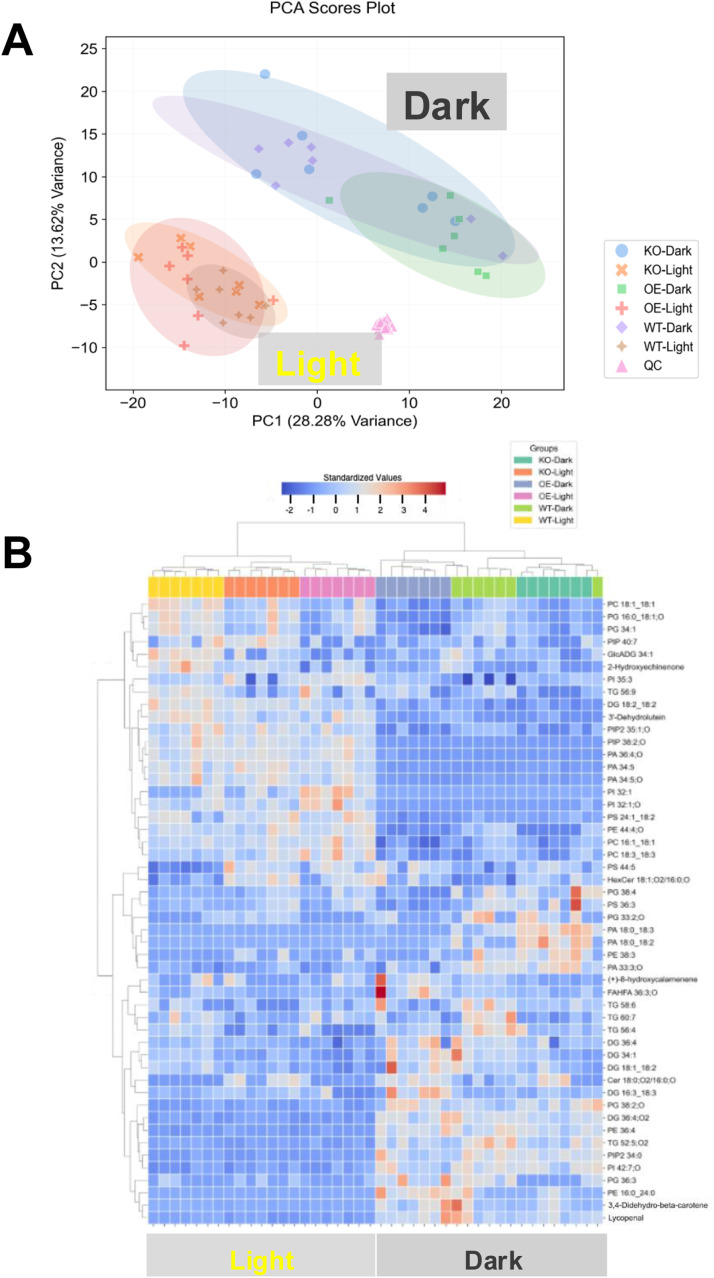
Distinct lipidomic profiles of light and dark-harvested *Arabidopsis* rosettes. A) Principal component analysis (PCA, PERMANOVA *F* = 6.55, *p* < 0.001) of auto-scaled, normalized peak intensities for annotated lipids shows separation of wild-type (WT), SLP1 knockout (KO), and over-expression (OE) samples by light condition. B) Clustered heatmap of the top 50 features ranked by Random Forest classification highlights condition and genotype-specific clustering. Intensities were normalized to internal standards and auto-scaled. Warmer colours (red) indicate higher relative abundance; cooler colours (blue) indicate lower abundance.

To evaluate global lipidomic shifts across conditions, we applied principal component analysis (PCA) using normalized and auto-scaled peak intensities for annotated features (Fig. 2A). The PCA scores plot displays tight clustering of QC technical replicates, indicating appropriate reproducibility and analytical consistency. This unsupervised method confirmed that light–dark transitions were the primary driver of lipidomic variation, separating all samples along the first principal component (PC1). In contrast, the three genotypes (wildtype - WT, SLP1 knockout - *slp1*-/-, and SLP1 over-expressor - OE) did not form distinct clusters. The absence of clear genotype separation indicates that loss or over-expression of SLP1 does not globally rewire the lipidome. Instead, its effects are likely restricted to specific lipid classes or species and may be masked in global PCA by the magnitude of the light–dark response. This suggests that the influence of SLP1 is subtle and operates through a limited number of downstream targets, rather than through wholesale remodelling of lipid metabolism.

To identify more localized lipid changes, we generated a heatmap of the annotated lipids across all samples (Fig. 2B). Lipid species were auto-scaled and clustered hierarchically to assess condition- and genotype-specific changes. Several lipid species, including specific PC, PA, phosphatidylglycerols (PG), and phosphatidylethanolamines (PE), show consistent shifts between SLP1 KO and OE, particularly under dark conditions. These trends are not class-wide but lipid-specific, reinforcing the conclusion that SLP1 alters a defined subset of lipid metabolic pathways.

The grouping of light versus dark and between each genotype illustrates the impact of the protein phosphatase SLP1 on lipid metabolism, as well as the dramatic influence of light-dark cycles on the lipidome. Compared to the wildtype, the three top-changing phospholipids were decreased in both SLP1 mutants, and the dark samples displayed lower values than the light samples. These initial differences displayed between lipidomes have led us to further pursue two lines of inquiry: 1) changes occurring between the light and dark lipidomes and 2) SLP1-mediated disturbances in the rosette lipidome.

### 3.3 Light and dark induce many alterations in the rosette lipidome

**Fig. 3:**
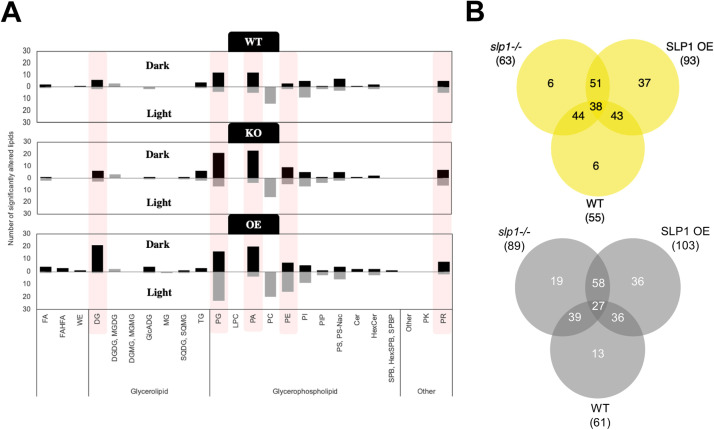
Light and dark conditions remodel lipidomic profiles of WT, *slp1-/-*, and SLP1 OE rosettes. A) Number of lipids significantly altered (fold-change ≥1.5 or ≤0.67, raw *p* < 0.05, FDR-*p* < 0.10). Bars represent lipids more abundant in dark (top, black) or light (bottom, gray). Groups showing marked genotype-specific differences are highlighted in pink. B) Venn diagrams indicate overlap in condition-dependent lipid changes across genotypes (Supplementary File S3). More lipids exhibit genotype-specific changes under dark conditions (bottom, grey) rather than under light (top, yellow).

Our data show that switching from a light to a dark environment confers extensive remodelling in the *Arabidopsis* lipidome (Fig. 3, Supplementary File S3). To identify individual lipid species significantly affected by SLP1 expression, we performed pairwise comparisons using Volcano plot analysis (Supplementary File S4). Differential abundance was assessed between genotypes (SLP1 knockout and over-expressor vs. WT) and between light and dark conditions. Over 10 lipid classes show species that diurnally increase or decrease by at least 1.5-fold in each genotype (Fig. 3A). Notably, the number of light-dark altered lipids in diacylglycerol (DG), PC, PA, and PE classes is over-represented across all genotypes. Prenol lipids also demonstrated light-dark enrichment of several species, with SLP1 OE having less PR enriched in the light than the WT or KO (Supplemental Table S1).

The light-dark transitions revealed 116 annotated lipids that changed significantly in wildtype rosettes, with a higher number changing in *slp1-/-* and nearly 100 additional lipids fluctuating in SLP1 OE plants (Fig. 3B**,** Supplemental Fig. S3). Among them, the putatively annotated PIP 38:2;O showed the most pronounced light-induced change across all three lines (Supplemental Fig. S4**,** Supplementary File S4). Because these ions yielded insufficient MS/MS signal for structural confirmation, annotations are based on accurate mass only and should be considered tentative. Hence, our interpretations involving these species are restricted to feature-level trends rather than structural confirmation. Still, the magnitude of light-dark abundance change surpasses 100-fold. Phosphatidyl inositol and especially phosphoinositides are low-abundance lipids in plant membranes, and the phosphorylated derivatives (PIP and PIP2) act as signaling molecules rather than bulk structural lipids [[46], [47], [48]]. Neutral extractions, such as those employed in this study, can retain small amounts of phosphorylated PI species, although recovery is substantially lower than with acidic protocols. Although PIP 38:2;O lacks MS/MS confirmation, the intensity, reproducibility, and specificity of the feature suggest a genuine biological change rather than an annotation artifact.

Other phosphatidylinositol species show a comparable pattern. PI 32:1, PI 32:1;O, PI 16:1_16:1 (PI 32:2), and PI 32:3 All increase 6 h into the light cycle across all genotypes, but to different extents: 3–7-fold for PI 32:1, over 10-fold for PI 32:1;O, 4–9-fold for PI 16:1_16:1 (PI 32:2), and 3–5-fold for PI 32:3. These trends were consistent across biological replicates and remained evident after internal-standard normalization and QC drift correction. PI 32:1;O shows a markedly larger light/dark ratio in WT and OE (46.6 and 51.7) than in *slp1-/-* (10.1). In contrast, the non-oxidized PI 32:1 displays higher fold-changes in *slp1-/-* (6.8) and OE (6.9) than in WT (3.0). The attenuated accumulation of PI 32:1;O in *slp1-/-* indicates that SLP1 influences (but does not strictly mediate) the light responsiveness of PI species. Given that oxidized and non-oxidized PIs rise in parallel, the data are consistent with coordinated regulation of PI pools during light exposure. These patterns may reflect changes in PI turnover or acyl editing, although the specific biochemical steps affected by SLP1 remain unresolved based on lipidomics alone. The light-dependent modulation of both oxidized and non-oxidized PI observed here reveals a novel layer of lipid remodelling during diel transitions.

Other oxidized lipids exhibit a consistent pattern of light enrichment across all genotypes (Supplemental Fig. S5). Interestingly, PI 34:2 increased 6 h into the light cycle, whereas the corresponding oxidized species PI 16:0_18:2;O and PI 16:0_18:2;O2 were not significantly altered (except for a PI 16:0_18:2;O2 form in the OE genotype). This pattern suggests that oxidized PI does not simply accumulate in parallel with its non-oxidized precursor. Instead, PI oxylipids likely belong to small, rapidly turned-over pools that are subject to active repair or degradation under light.

Lutein B, a xanthophyll derived from the chloroplast MEP pathway, was significantly increased 6 h into the light cycle across WT, *slp1-/-*, and SLP1 OE (Supplementary File S4). Lutein B has a functional role in quenching singlet oxygen and dissipating excess excitation energy within the chloroplast, primarily acting as a photoprotective antioxidant in plants. Its light enrichment in WT and mutant lines supports the interpretation of elevated chloroplast redox activity or enhanced need for antioxidant buffering.

Although total prenol lipid abundance was not significantly different across genotypes or light conditions (Supplemental Fig. S6), individual species, especially those linked to chloroplast redox status, showed SLP1-dependent variation. This supports a model in which SLP1 modulates specific redox-sensitive pathways or plastid metabolite export, rather than acting on bulk isoprenoid synthesis.

Among the most strongly dark-enriched features, diacylglycerol DG 32:0 accumulated nearly 10-fold in the dark across all genotypes (Supplementary File S4). DGs are intermediates in galactolipid biosynthesis and triacylglycerol (TG) and phospholipid turnover, and their increase in darkness is consistent with reduced flux through MGDG and DGDG synthesis. This interpretation is supported by the concurrent depletion of plastidial galactolipids, such as MGDG 16:0_18:3 and MGDG 16:1_16:3, suggesting a shift away from membrane biogenesis 6 h into the dark cycle. Notably, multiple PCs with high-confidence annotations (e.g., PC 16:0_18:1, PC 16:1_18:1, PC 18:0_18:1) also showed consistent and significant depletion in the dark, whereas several PA species increased, consistent with extraplastidic phospholipid remodeling [28]. The broad reduction across both plastid and extraplastidic phospholipids, together with DG accumulation, reflects a coordinated metabolic shift away from membrane assembly during the dark period.

Several other DG species, including oxidized forms such as DG 36:2;O2, DG 36:4;O2, and DG 36:5;O2, also increased significantly in the dark, indicating a broader shift in glycerolipid turnover rather than a species-specific effect. In addition, TG 52:5;O2, an oxidized triacylglycerol species, showed consistent dark-phase enrichment across genotypes. The accumulation of oxidized glycerolipids in darkness is counterintuitive, given that ROS production is primarily light-dependent. This suggests these lipids are unlikely to be direct markers of oxidative stress. Instead, their increase may arise from regulated lipid remodeling processes, such as such as lipase-mediated degalactosylation, PA→DG cycling, or turnover of PUFA-rich membrane lipids, combined with low-level, non-enzymatic peroxidation that persists at night. Enzymatic oxidation cannot be excluded, but specific pathways remain undefined. In contrast, the only high-confidence DG with a clear light-dominant pattern, DG 18:2_18:2, was strongly light-enriched, highlighting distinct metabolic or compartmental origins for different DG pools. The consistent dark-driven increase in DG 32:0 and oxidized DGs across WT, KO, and OE lines further supports the idea that this response is governed by core diel regulatory programs, rather than an SLP1-dependent effect [49,50].

Several high-confidence annotated species showed strong and reproducible diurnal changes consistent with known shifts in plastid lipid metabolism. Previous studies have reported increased total trienoate (18:3, 16:3) abundance at the end of a 12 h dark cycle, quantified at the level of total fatty-acid pools or class-level FAME profiles, reflecting continued desaturation during the night [[26], [27], [28]]. Our samples, however, were collected six hours into an eight-hour dark cycle, representing a different metabolic phase than end-of-night sampling. At this mid-dark time point, we observed significantly elevated levels of PA 18:0_18:3, PS 24:1_18:3, PG 16:1_16:3, and several polyunsaturated phosphatidic acid and glycerolipid species. In contrast, free FA 18:3, an intermediate for oxylipin synthesis and plastid membrane lipids, was more than threefold higher in the light across all genotypes. This pattern is consistent with membrane remodelling and phospholipase D (PLD) activity and differs from the nocturnal enrichment in total trienoate fatty acids reported by end-of-cycle FAME-based studies.

Individual molecular species are regulated through distinct turnover, remodeling, and acyl-editing processes. Consistent with this, Maatta et al. (2012) report that MGDG, PG, and PC classes contain species that peak in the light and others that peak in the dark [28]. Our data reflect this species-level heterogeneity: several polyunsaturated species increase during the light period, whereas others show higher abundances at mid-dark. Notably, several 18:3-rich structural lipids, including MG 16:1_16:3, MGDG 16:0_18:3, PC 18:3_18:3, and free FA 18:3, decreased in the dark, while most PA species increased. This pattern is consistent with a model in which dark-phase activation of enzymatic reactions drives PA accumulation from trienoate-containing glycerolipids. In this scenario, MGDG and PC molecular species, together with free 18:3, serve as substrates for PA production and signaling rather than forming bulk trienoate pools. Our 16/8 photoperiod and mid-dark sampling, therefore, capture a phase in which 18:3 is being mobilized through PA rather than the end-of-night trienoate enrichment described in prior diel FA studies.

Diurnal changes were also evident in lipid structural features, particularly acyl tail saturation and carbon number (Supplemental Fig. S7**,** Supplemental Fig. S8). Light conditions favoured both even- and odd-chain acyl tails depending on genotype, with odd-chain enrichment generally more prominent in the dark (Supplemental Fig. S7)**.** Across all genotypes, mono- and polyunsaturated chains were reduced in the dark, while saturated acyl tails were relatively enriched, especially in the OE background (Supplemental Fig. S7). Other classes, including PS, PI, and PG, showed more modest diurnal shifts in acyl chain length. In SLP1 OE, we observed altered light-dark transitions in specific lipid groups, such as increased 52C TG and decreased 60C TG in the light(Supplementary File S4), consistent with broader disruption of lipid elongation or remodelling.

### 3.4 Characteristics of SLP1 mutant lipidomes

**Fig. 4:**
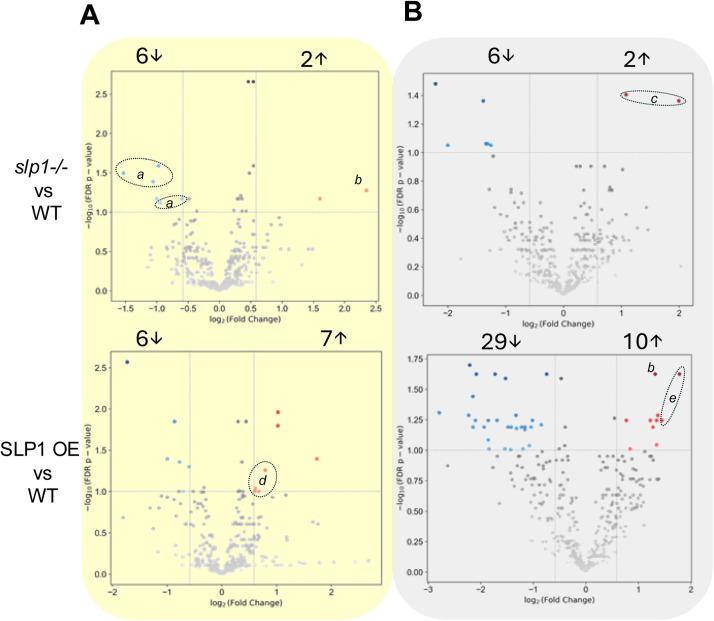
SLP1 mutant lipidomes differ from WT lipidomes in the light and dark. Volcano plots show lipids with significantly different abundances (fold-change ≥1.5 or ≤0.67, raw *p* < 0.05, FDR-*p*< 0.10) in the knockout (*slp1-/-*) or over-expression (SLP1 OE) lines compared to wildtype (WT) in A) light (yellow) or B) dark (gray). Numbers above each panel indicate the count of significantly altered lipid species. Lipids of particular interest are labelled: a- triacylglycerols, b- ceramide Cer 18:0;O2/16:0;O (18:0 dihydroxy sphingoid base and 16:0 hydroxylated acyl chain), c- phosphatidic acids, d- phosphatidylcholines, e- phosphatidylinositols.

In contrast to the broad lipidomic remodelling triggered by light-dark transitions, aberrant expression of SLP1 induces a more selective and attenuated response in the rosette lipidome. Comparisons between *slp1-/-* and WT reveal relatively few significant changes in either light or dark conditions, and SLP1 OE shows a similar limited shift under light (Fig. 4). However, in the dark, SLP1 OE plants exhibit a more pronounced lipidomic divergence, with 39 species showing significant alterations. Despite fewer changes, both mutants maintain distinct and reproducible lipidomic profiles.

In *slp1-/-*, several triacylglycerols (TG 56:4, 56:7, 56:9, 57:6, and 59:6) are significantly reduced under light, a pattern only partially reproduced in the dark, where the strongest difference is increased phosphatidic acid (PA 18:0_18:2 and PA 18:0_18:3) (Fig. 4). SLP1 OE plants, in contrast, accumulate several 18:3-containing phospholipids in the light (PC 16:0_18:3, PC 18:2_18:3, PC 18:3_18:3, and PE 18:0_18:3), consistent with enhanced desaturation or stabilization of polyunsaturated membrane species. This enrichment is not maintained during the dark period, where phospholipids are broadly depleted (Supplemental Fig. S9) and diacylglycerol DG 16:3_18:3 is significantly increased (Supplementary File S4). PA 18:0_18:3, increased in the light in SLP1 OE, is conversely decreased in the dark, suggesting a coordinated shift in plastidial lipid synthesis and remodeling.

The behaviour of 18:3-containing lipids provides mechanistic clues into the diel and SLP1-dependent remodeling of membrane lipids. α-Linolenic acid (18:3) is the terminal product of plastidial and ER-localized desaturation and a major component of thylakoid membranes, where its polyunsaturation supports membrane fluidity and photosystem function [51]. Its biosynthesis is light-enhanced and suppressed in darkness, consistent with known regulation of desaturases such as FAD7, FAD8, and FAD3 [52]. In our data, FA 18:3, MGDG 16:0_18:3, and PC 18:3_18:3 are consistently enriched in the light and depleted in the dark across genotypes, indicating light-driven desaturation and/or stabilization of polyunsaturated lipids. This diel cycling is attenuated or distorted in SLP1 mutants. SLP1 OE accumulates additional 18:3-PC species under light, whereas the KO exhibits reduced light-phase TGs and PA, and a blunted DG diurnal response, suggesting impaired lipid remodeling, export, or acyl editing. The opposite effects in KO and OE support the hypothesis that SLP1 influences either desaturase regulation, ER–plastid lipid exchange, or downstream acyl turnover mechanisms.

Previous studies have reported increased total trienoate (18:3, 16:3) abundance at night, quantified at the level of total fatty-acid pools or class-level FAME profiles, rather than individual lipid molecular species [[26], [27], [28]]. These FA-level dynamics reflect ongoing desaturation during the dark period but do not directly predict the behavior of each molecular species, which are regulated through distinct turnover, remodeling, and acyl-editing processes. Several polyunsaturated molecular species increase under light conditions, consistent with class-specific turnover and acyl-editing dynamics rather than bulk FA enrichment alone.

**Fig. 5:**
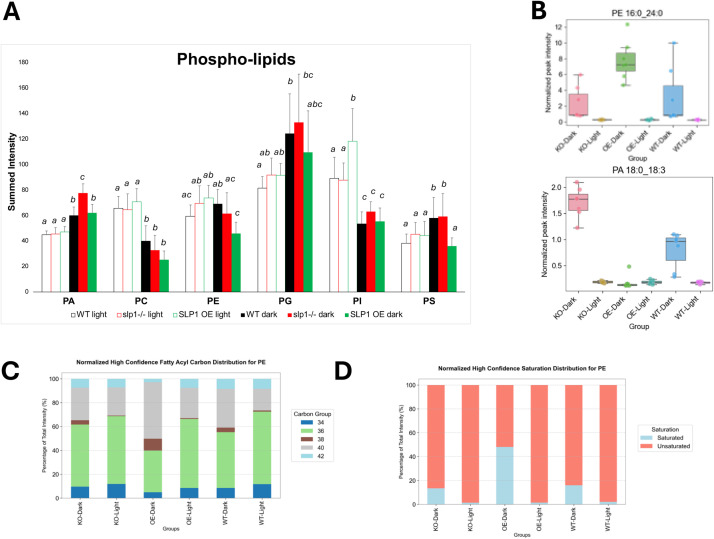
Phospholipid abundance and saturation in *Arabidopsis* rosettes vary with light conditions and SLP1 expression. A) Summed intensity for each phospholipid subclass in WT and SLP1 mutants under light and dark conditions. Bars represent mean values; error bars show standard deviations. One-way ANOVA followed by Tukey’s Honestly Significant Difference test (*p* < 0.05) was used to assess differences among groups. Within each phospholipid subclass, groups sharing the same letter are not significantly different from each other. B) Box plots show the abundance of two representative high-confidence annotated lipids to exemplify the differences in PA and PE levels between WT and SLP1 mutants. C-D) In SLP1 OE plants under dark conditions, altered PE metabolism is reflected in differences in C) carbon chain length and D) acyl tail saturation for high-confidence PE lipids.

Phospholipid class behaviour was further resolved by examining total subclass intensities (Fig. 5A). WT and *slp1-/-* plants exhibit minimal total phospholipid changes across diel cycles, but SLP1 OE shows a pronounced drop in phospholipid abundance from light to dark (Supplemental Fig. S9**,** Fig. 4B). In WT, PA, PG, and PS increase in the dark, while PC and PI are enriched in the light. These general trends are retained in the mutants with one notable exception: PS. Only *slp1-/-* retains the WT pattern of dark-phase PS increase, while PS is markedly lower in SLP1 OE in the dark. Elevated PA in *slp1-/-* dark samples aligns with the PA species identified in the volcano plot (Figs. 4B **and** Fig. 5B), suggesting SLP1 influences PA turnover or synthesis.

PI trends are more complex. Total PI is significantly increased in the light in SLP1 OE (Fig. 5A). Yet, several individual PI species accumulate specifically in the dark (e.g., PI 33:3, 35:1, 36:1). This decoupling of total and individual lipid behaviour suggests altered acyl chain remodelling or selective turnover within the PI pool, rather than uniform upregulation.

SLP1 OE also introduces a unique diurnal fluctuation in phosphatidylethanolamine. Although total PE abundance in SLP1 OE is lower than WT in the dark (Fig. 5A), the acyl composition is markedly shifted toward longer and more saturated tails (Fig. 5C). PE 16:0_24:0, a high-confidence annotated lipid, exemplifies this shift (Fig. 5B and D), indicating that SLP1 may influence PE biosynthesis or remodelling in a chain-length- and saturation-dependent manner.

**Fig. 6:**
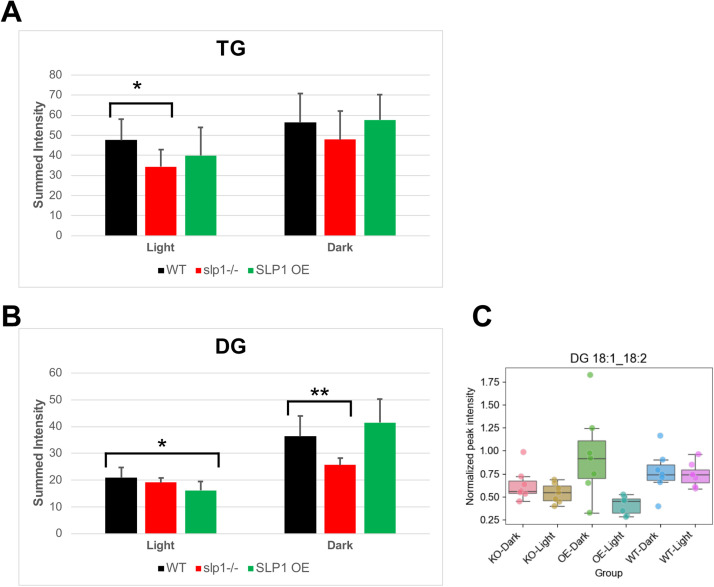
Triacylglycerol (TG) and diacylglycerol (DG) abundance in WT and SLP1 mutant rosettes. A-B) Summed intensities for A) TG and B) DG species across WT, SLP1 knockout (*slp1-/-*, KO) and SLP1 overexpression (OE) under light and dark conditions. Bars show mean ± one standard deviation. Student’s *t*-tests were used to assess differences between light and dark within and between genotypes. *p < 0.05, **p < 0.01. C) Box plot depicting the abundance of a representative DG lipid annotated with high confidence, illustrating species-specific trends consistent with the summed DG contributions.

Triacylglycerol metabolism is also affected. TGs are significantly depleted in the light in *slp1-/-*, both at the species level (Fig. 4A) and total class intensities (Fig. 6A). Most of the affected TGs in *slp1-/-* are long-chain, polyunsaturated, and even-numbered (Supplemental Figure S10), suggesting altered storage lipid utilization or biosynthesis. Given that DG is a precursor for TG synthesis, we also analyzed total DG levels (Fig. 6B), which rise in the dark in all genotypes, increasing approximately 75 %, 35 %, and 150 % in WT, *slp1-/-*, and SLP1 OE, respectively (Fig. 6B). The exaggerated dark-phase accumulation in SLP1 OE corresponds with the increase in specific dark-enriched DGs (e.g., DG 18:1_18:2, Fig. 6C). This pattern suggests SLP1 modulates DG metabolism, potentially affecting both biosynthetic flux and acyl remodelling.

Finally, one ceramide species exhibits opposing diel accumulation in the two SLP1 mutants (Cer 18:0;O2/16:0;O, Fig. 4**,** label *b*), suggesting a role for SLP1 in sphingolipid metabolism or signalling compartmentalization. Although isolated, this reciprocal behaviour further supports the broader conclusion that SLP1 influences specific lipid subnetworks through both gain and loss-of-function perturbations.

## 4 Discussion

The chloroplast is a metabolically active organelle that supports photosynthesis, carbon fixation via the Calvin cycle, and the *de novo* biosynthesis of fatty acids and lipids [[53], [54], [55]]. Protein phosphorylation is a widespread regulatory mechanism in plastids [[56], [57], [58]], but the specific targets, protein kinases, and phosphatases remain poorly defined. SLP1 localizes exclusively to the chloroplast; however, its endogenous substrates remain unknown. To investigate its physiological role, we employed quantitative lipidomics to profile *Arabidopsis* rosettes lacking SLP1 (*slp1-/-*) and those over-expressing it (SLP1 OE). Both mutants exhibit distinct lipidomic alterations, implicating SLP1 in the regulation of chloroplast lipid metabolism and in processes that affect whole-cell lipid composition. Given its function as a protein phosphatase, we hypothesize that SLP1 modulates lipid flux or remodelling indirectly by dephosphorylating chloroplast proteins involved in lipid biosynthesis, trafficking, or export. Below, we highlight several lipidomic changes that support this hypothesis.

### 4.1 Light and dark lipidomic profiles in *Arabidopsis* rosette

Multiple prenol lipids annotated in our dataset are derived from the isoprenoid biosynthetic network, which in plants involves both the MEP and mevalonate (MVA) pathways. The MEP pathway, localized in plastids, is subject to transcriptional regulation by the circadian clock and light-responsive signals (reviewed in [59,60]). Consistent with this, plastochromenol levels fluctuate across the light-dark cycle in all genotypes. This may reflect dysregulation of plastidial isoprenoid metabolism, although a direct mechanistic link remains undefined.

Fatty acyl saturation patterns also shift between light and dark conditions, in agreement with prior studies reporting plastid desaturase activity as light-sensitive [11,28]. One notable and potentially novel feature in our data is the selective enrichment of odd-chain fatty acids (OCFAs) in the dark. To our knowledge, diurnal regulation of OCFAs has not been previously documented in *Arabidopsis*. Given the metabolic interest in OCFAs for both biotechnological and signalling roles, this diel enrichment warrants further investigation into their biosynthetic origin and regulation [[61], [62], [63], [64], [65], [66], [67]]. Because most OCFA assignments in these datasets are mass-only or low-fragmentation-confidence species, we interpret this diel trend cautiously and present it as a candidate phenomenon rather than a validated metabolic pathway.

Another prominent feature is the elevated abundance of oxidized lipid species in the light relative to the dark. Lipid peroxidation is a well-established consequence of reactive oxygen species (ROS) produced from biotic or abiotic stress, e.g. infection, heat, and high light. Chloroplasts are a major source of ROS during photosynthetic electron transport, particularly under high light, and polyunsaturated fatty acids such as 18:3 are especially susceptible to peroxidation [68]. However, ROS are produced even in low light conditions (similar to growth light in this work), albeit at a much lower level [69]. Future work could investigate the physiological significance of oxylipin abundance cycling from light to dark. Counterintuitively, some oxidized lipids in our dataset are dark-enriched [70]. This may suggest contributions from enzymatic oxidation, particularly via lipoxygenases such as LOX2, which is localized in the chloroplast stroma and shows light-independent activity [71]. The presence of oxidized PG, a lipid of chloroplast origin, is consistent with both ROS-driven and enzymatic mechanisms.

Our data reinforce previously described diel shifts in several phospholipid classes [28,72]. These include light-enriched PC and PI species, and dark-enriched PA and PS species. Some of these trends are consistent with transcript-level evidence for diurnally regulated enzymes in phospholipid metabolism; for example, phospholipase D (PLD) transcripts show a strong diurnal profile, whereas phosphatidylserine decarboxylase 3 (PSD3) transcripts show both diurnal and circadian dependence [73,74]. These changes are further modulated in SLP1 mutants, suggesting SLP1 contributes to the regulation of chloroplast-derived lipid export or remodelling in a light-dependent context. Specific lipids affected in *slp1-/-* and SLP1 OE, such as PA 18:0_18:3, PS 18:0_18:2, and multiple 18:3-containing PCs, support this interpretation.

### 4.2 Regulation of lipid metabolism by SLP1

**Fig. 7:**
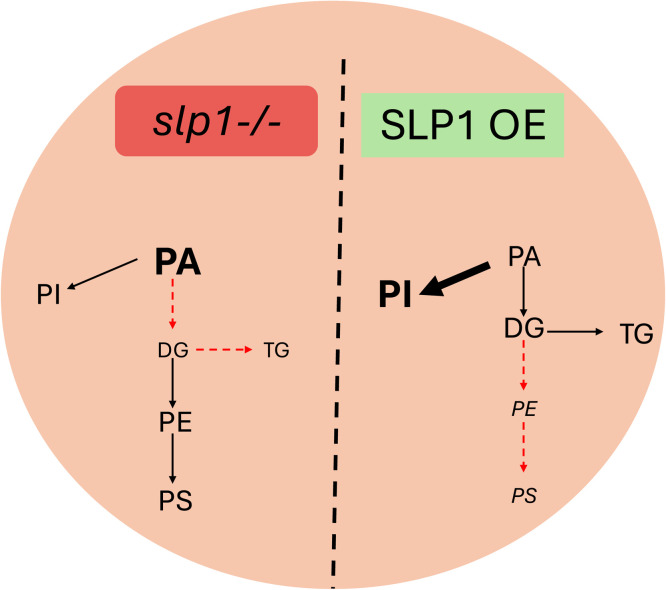
SLP1 plays a role in *Arabidopsis* rosette glycerolipid metabolism. In *slp1-/-*, elevated phosphatidic acid (PA) levels may contribute to the observed decrease in triacylglycerol (TG) abundance. In contrast, SLP1 over-expression (OE) leads to the accumulation of phosphatidylinositols (PI), which may hinder the synthesis of phosphatidylethanolamine (PE) and phosphatidylserine (PS) lipids. Font sizes indicate relative lipid abundances. Red dashed lines are used to postulate affected metabolic pathways.

Our data demonstrate that SLP1 has a significant impact on phospholipid and neutral lipid homeostasis, indicating a role in regulating glycerolipid metabolism in *Arabidopsis*. In *slp1-/-*, the accumulation of phosphatidic acid is a prominent feature and represents congestion of an important crossroads in the plastid. PA is a metabolic intermediate linking fatty acid synthesis to the production of structural and storage lipids, including DG, TG, and phospholipids. Interestingly, Kim et al. postulate that PA’s in vitro binding ability to elements of the circadian clock are responsible for regulating TAG levels [75,76]. Our data are consistent with this model, showing increased PA in *slp1-/-* and reduced TG levels (Fig. 7). The selective accumulation of PA in *slp1-/-,* without a corresponding rise in downstream phospholipids, may indicate impaired conversion into DG or dysregulation of enzymes downstream of PA, such as PAP or DGAT. Correspondingly, TG levels are reduced in *slp1-/-*, supporting this interpretation. Whether light-dark oscillations in lipid intermediates influence subsequent diel cycles remains unresolved; however, the accumulation of DG in SLP1 OE in the dark implies potential metabolic carryover across periods. This possibility deserves further attention in future time-course experiments.

In SLP1 OE, the lipid profile is characterized by altered phospholipid levels, specifically reductions in most phospholipids and increases in PI 6 h into the dark cycle, indicating a shift in flux toward PI biosynthesis or a selective loss of PE/PS stability under altered phosphorylation states. Because PE and PS are interconverted through a decarboxylation step, their concurrent reduction in SLP1 OE during the dark may indicate suppression of a shared synthetic node. The concurrent rise in PI suggests redirection of cytidine diphosphate–diacylglycerol (CDP-DAG) intermediates away from PE/PS branches, although this possibility remains speculative and requires confirmation. This altered flux might result from post-translational regulation of enzymes in the Kennedy pathway or its plastidial equivalents, potentially through SLP1-mediated dephosphorylation of rate-limiting steps.

Given the known tissue-specific roles of glycerolipids, future lipidomic profiling of multiple organs would be necessary to establish whether the observed changes in rosettes are generalizable. For instance, PCs enriched in SLP1 OE rosettes during the light period resemble species previously linked to early flowering in *Arabidopsis* meristem tissue [72]. This correspondence may explain the early flowering phenotype observed in SLP1 OE (unpublished observation), but such a connection remains speculative. Similarly, since TG content is reduced in *slp1-/-* rosettes, it is plausible that seed TG reserves are also diminished. This hypothesis is of particular interest given the commercial focus on maximizing seed oil content.

SLP1 also appears to modulate the diel homeostasis of multiple phospholipids, with altered class-wide dynamics between light and dark periods. This reprogramming could change membrane architecture and dynamics over light-dark cycles, given the structural roles of PA and PS in the bilayer. PA, a cone-shaped lipid, tends to promote negative membrane curvature and is implicated in vesicle budding, membrane fusion, and organelle biogenesis [77]. PS, while less conical than PA, also contributes to membrane curvature and provides electrostatic surfaces for protein recruitment, particularly at membrane contact sites and signaling hubs [78].

Moreover, arabidopsides, a class of oxidized galactolipids associated with chloroplast stress responses, are significantly enriched in SLP1 OE during the dark. Arabidopsides are stress-inducible lipids, so their dark-phase enrichment in SLP1 OE may happen due to altered plastid stress signalling or turnover rather than direct enzymatic regulation by SLP1. Arabidopsides contain oxylipins derived from the LOX2-mediated oxidation of monogalactosyldiacylglycerol (MGDG) and digalactosyldiacylglycerol (DGDG) [79,80], and are typically produced in response to biotic and abiotic stresses such as pathogen attack, wounding, and high light [81,82]. Their accumulation in SLP1 OE plants suggests that SLP1 overexpression triggers or amplifies chloroplast stress signaling pathways, potentially by altering the redox state or enzymatic activities within the plastid. This increase in arabidopsides may also reflect a heightened defense state in the chloroplast, possibly linking SLP1 activity to lipid-mediated stress signaling [83]. Intriguingly, trienoic acids have also been shown to have a plant defense role [84,85], and here we have demonstrated that these 18:3 lipids accumulate with SLP1 over-expression. Lastly, increased arabidopsides could also arise from disrupted degradation or remodelling of plastid lipids in the absence of regular phosphatase control. Distinguishing between these three outlined possibilities will require follow-up stress marker profiling and kinetic lipid flux analysis.

The extensive disruptions observed in both SLP1 gain- and loss-of-function lines suggest that this protein phosphatase broadly influences lipid metabolism in *Arabidopsis*, likely through signaling cascades initiated by its action on chloroplast-localized target proteins. Possible substrates include enzymes within the fatty acid synthesis pathway, such as acetyl-CoA carboxylase (ACCase) or its regulators (e.g., BADC proteins), as well as FA exporters like the FAX proteins [86]. Many of these proteins are phosphoproteins according to large-scale phospho-proteomic datasets [57,87], supporting their candidacy as SLP1 targets. Nevertheless, not all lipid changes observed in the SLP1 mutants can be explained by plastid-localized processes. Cytosolic or ER-based lipid remodelling may be indirectly affected through retrograde signalling or changes in energy/nutrient status. For instance, preliminary unpublished data from our group shows altered sugar levels in the same SLP1 mutants in this work; altered sugar levels can modulate the Target of Rapamycin (TOR) signalling pathway, which is known to regulate lipid biosynthesis and membrane biogenesis in plants [88].

Previous *Arabidopsis* lipidomics work has mainly focused on defining steady-state compositions of leaf and chloroplast lipids, stress-induced remodeling, or the effects of mutations in lipid biosynthetic enzymes [[26], [27], [28]]. Diurnal changes in lipid classes and acyl profiles have been reported, and phosphoproteomic studies have mapped extensive chloroplast protein phosphorylation, yet these datasets have rarely been integrated to ask how plastid phosphatases shape lipid outputs over the light–dark cycle. Our results show that perturbing a single chloroplast phosphatase is sufficient to alter specific branches of glycerolipid and PI metabolism and to modify the diel behaviour of oxidized lipids, without collapsing the global galactolipid-rich architecture of the *Arabidopsis* leaf lipidome. In that sense, SLP1 connects the well-established chloroplast phosphorylation network to measurable shifts in membrane and signaling lipids across organelles, linking plastid protein dephosphorylation to whole-cell lipid homeostasis under a physiologically relevant light–dark regime.

### 4.3 Methodological limitations

This study used an untargeted lipidomics workflow to investigate the regulatory role of SLP1 in *Arabidopsis thaliana*. Future work may use targeted assays to quantify selected SLP1-responsive lipids, but the unbiased nature of untargeted profiling was essential for identifying these candidates in the first place. We aimed to determine relative lipid changes across the investigated models, not to reconstruct absolute lipid abundances or provide targeted quantification of predefined species. Accordingly, we focus on reproducible, condition-dependent changes rather than absolute lipid abundances or exhaustive lipid recovery. The freeze–grind procedure used here for lipid extraction from *Arabidopsis* rosettes can alter levels of PA, DG, and oxidized galactolipids if tissue is allowed to thaw prior to solvent quenching [[89], [90], [91]]. Hence, samples were immediately extracted in a cold room using chilled solvents to minimize disturbances. All genotypes and light–dark conditions were processed identically to ensure that relative comparisons across lines remain valid. The data described in this work are normalized intensities rather than raw peak areas or molar concentrations (i.e., relative quantifications). Untargeted lipidomics requires appropriate normalization strategies to correct for experimental variations. Normalized intensities allow statistically valid comparisons between experimental groups, but they should not be interpreted as the true stoichiometric distribution of lipid species within *Arabidopsis* tissue. We therefore note that our normalized signal intensities cannot be directly compared to published absolute mol % lipid compositions, as the datasets measure fundamentally different quantitative endpoints.

Untargeted lipid annotation in this study was performed using accurate mass, MS/MS fragmentation, and retention times (RT), complemented by biological plausibility. Among these, RT filtering is essential for reducing false-positive assignments in complex matrices, where structural isomers, in-source fragments, and adduct redundancies are frequent. RT windows restrict annotations to elution intervals that are chemically plausible for each lipid class, improving spectral specificity and reducing misidentification of isobaric species. This step is widely used and necessary for annotation accuracy in high-throughput lipidomics. However, RT-based filtering inevitably introduces bias. RT boundaries must be defined empirically or predicted from similar standards, and in this study, they were optimized without *Arabidopsis*-specific calibration. We recognize that some bona fide *Arabidopsis* lipids, such as highly unsaturated or oxidized MGDG, DGDG, plant PI species, and selected sphingolipids, eluted outside the RT windows used during initial processing and were therefore excluded from annotation. These features are present in the raw MS data but remain in the “unknown” pool (Supplementary File 2 - Table 2) for the processed data discussed in this manuscript. Given the number of detected features, comprehensive manual correction was not feasible. The comparisons presented here are therefore based on a consistent subset of the rosette lipidome, rather than a comprehensive reconstruction of total lipid composition.

To demonstrate that these major *Arabidopsis* lipids were detected but filtered out, we plotted representative features mass-matched to MGDG and DGDG species (Supplemental Fig. S11). These features show robust signal and reproducible light–dark trends across samples, although they were not significantly altered in the investigated KO, OE, and WT states. MGDG 36:8;O2 was an exception, being significantly elevated for KO/OE (dark cycle). Their absence from the annotated, normalized tables reflects RT filtering rather than biological deficiency. Comprehensive plant lipidomes have been characterized extensively in previous studies. Here, all samples were processed uniformly with identical annotation rules, so genotype- and light–dark comparisons remain internally valid, even though the absolute class proportions are incomplete. Future work using plant-specific RT libraries or RT prediction models will reduce this bias.

A second limitation relates to the inability of Orbitrap LC-MS/MS to resolve structural isomers, including positional isomers, functional-group isomers, and stereoisomers. Many lipid species share identical chemical formulas and nominal masses and often produce highly similar MS/MS fragmentation patterns. As a result, this untargeted workflow cannot determine double-bond positions, stereochemistry, or regioisomeric acyl-chain arrangement with confidence. This limitation also explains occasional misannotations of canonical species. For example, PC 16:0_18:2 (PC 34:2) may be assigned to isobaric alternatives, such as PC 16:1_18:1 or PC 17:1_17:1. These compositions have identical nominal masses and may be co-isolated for fragmentation or lack sufficiently diagnostic MS/MS fragments to distinguish them in the absence of targeted validation or specialized structural workflows. Furthermore, a few annotated species with unexpected very-long-chain, highly unsaturated acyl tails (e.g., a TG containing a 24:5 acyl chain) may represent mass-only misassignments or isobaric/in-source artifacts rather than genuine *Arabidopsis* lipids. These features are retained in the supplemental data for completeness but are not used for mechanistic interpretation and should be regarded as tentative assignments.

Whole-rosette lipidomics introduces biological heterogeneity due to tissue composition and developmental variation, contributing to intra-group spread in some lipid species. In addition, regulatory enzymes such as protein phosphatases rarely produce inverse phenotypes when knocked out or overexpressed; the relationship between phosphorylation state and metabolite output is nonlinear. Therefore, KO and OE lines are not expected to show mirror-image lipid changes. Our conclusions rely on reproducible, pathway-level trends rather than symmetric effect sizes, and these patterns remain internally consistent across replicates and diel conditions.

## 5 Conclusion

The chloroplast-localized phosphatase SLP1 influences plant lipid metabolism and contributes to diel lipid remodeling. Altering SLP1 activity perturbs specific branches of glycerolipid and phosphoinositide metabolism within the plastid and produces coordinated changes in extraplastidic lipid pools, indicating cross-compartment communication. These findings place SLP1 within the broader regulatory network that links chloroplast protein phosphorylation to whole-cell lipid homeostasis. The data support a model in which SLP1 modulates the phosphorylation states of enzymes involved in lipid biosynthesis, turnover or transport. Defining the direct substrates of SLP1 will be required to resolve the underlying biochemical pathway and to understand how plastid dephosphorylation events propagate through cellular lipid networks under physiologically relevant light–dark cycles.

## Funding

Lipidomics data were acquired at the Calgary Metabolomics Research Facility (CMRF) through the Buzatto Research Group. GM and AZB are funded by the Natural Sciences and Engineering Research Council of Canada (NSERC). This research was undertaken, in part, thanks to funding from the Canada Research Chairs Program to AZB.

## Consent for publication

All authors consent to publication.

## Data availability

The lipidomics data described in the article can be found in the supplementary files S1-S4. The raw data have been deposited in MetaboLights repository with the study identifier MTBLS13371 [38].

## CRediT authorship contribution statement

**Chris White-Gloria:** Writing – original draft, Visualization, Investigation, Formal analysis, Data curation. **Greg B. Moorhead:** Writing – original draft, Supervision, Project administration, Funding acquisition, Conceptualization. **Adriana Zardini Buzatto:** Writing – original draft, Visualization, Supervision, Funding acquisition, Formal analysis, Data curation, Conceptualization.

## Declaration of competing interest

The authors declare that they have no known competing financial interests or personal relationships that could have appeared to influence the work reported in this paper.

## Glossary

FA: fatty acids
FDR: false discovery rate
GL: glycerolipids
GP: glycerophospholipids
KO: knockout line, *slp1-/-*
MEP: methylerythritol phosphate
LC-MS/MS: liquid chromatography tandem mass spectrometry
OCFA: odd chain fatty acids
OE: over-expressor line, SLP1 OE
PCA: principal component analysis
PLS-DA: partial least squares discriminant analysis
PPP: phosphoprotein phosphatase
PR: prenol lipid
QC: quality control
ROS: reactive oxygen species
SLP1: *Shewanella*-like protein phosphatase 1
SP: sphingolipids
ST: sterols
WT: wild-type

## Appendix Supplementary materials

Supplemental Figure S1. Lipid class distribution of annotated peaks from untargeted lipidomics of *Arabidopsis* rosette tissue. High confidence lipids (117) were annotated based on MS/MS spectral matches with a score ≥ 400 and *m/z* error ≤ 2.5 ppm or 3.0 mDa). Low confidence annotations (301) were assigned based on mass matches within *m/z* error of 2.5 ppm. All annotations passed retention time, adduct, ionization patterns, and biological context filtering. Abbreviations: FA- fatty acids, GL- glycerolipids, GP- glycerophospholipids, SP- sphingolipids, ST- sterols. Additional abbreviations for lipid classes and subclasses are listed in Supplemental Data 1. Image, application 1

Supplemental Figure S2. Annotated lipids with the 30 top raw intensities in wild-type plants during light and dark cycles. * p (Light/Dark) <0.05, ** p (Light/Dark) <0.05 and fold-change (Light/Dark) ≤0.667 or ≥1.50. Image, application 2

Supplemental Figure S3. SLP1 influences the differential diurnal enrichment of lipids in *Arabidopsis* rosettes. A) Volcano plot analysis reveals significantly altered lipids for light and dark conditions (fold-change ≥1.5 or ≤0.67, raw *p* < 0.05, FDR-*p* < 0.10) within each *Arabidopsis* genotype. A complete list of lipid annotations, fold-changes, and p-values is provided in Supplemental Data 2. Selected lipids of particular interest are labelled as: a- PIP 38:2;O, b-PI 32:1;O, c-DG 32:0. B) Lipid set enrichment analysis (LSEA) indicates that long-chained, even carbon-numbered, polyunsaturated phosphatidylglycerol species are specifically enriched in *slp1-/-* dark rosettes. LSEA was performed using a custom Python script that applies a modified gene set enrichment logic to lipidomics data. Lipid species were grouped into sets based on shared structural features (e.g., headgroup, chain length, saturation, or subclass). Enrichment was assessed using a one-sided Fisher’s exact test comparing significantly altered lipids (fold change ≥1.5, *p* < 0.05, FDR < 0.10) against the background of all detected lipids. Lipid sets with FDR-adjusted *p* < 0.10 were considered significantly enriched. Image, application 3

Supplemental Figure S4. Phosphatidylinositol phosphates (PIP) show diurnal abundance and saturation changes in *Arabidopsis* rosettes. A) Violin plot depicting the variation in summed intensity of PIPs under light and dark conditions for all three genotypes. B) Proportion of PIP peak intensities with (un)saturated carbon tails across light and dark conditions in SLP1 wild-type (WT), knockout (KO, *slp1-/-*), and over-expression lines (OE). Supplemental Data 1 lists all PIP lipid annotations used for analyses. C) Image, application 4

Supplemental Figure S5. Total abundances of oxidized lipids in *Arabidopsis* rosettes fluctuate diurnally. A) Summed intensities for oxidized glycerolipids, glycerophospholipids, and fatty acyls for light and dark in WT and SLP1 mutant lines. The bars represent mean values ± one standard deviation. B) The phosphatidylglycerol PG 16:0_18:2;O2 represents a light-enriched oxylipid. C) Unsaturated phosphatidylinositols (PI) with 34 carbons exhibit distinct light–dark behaviours. PI 34:2 is strongly enriched in the light across all genotypes. Likewise, the oxidized species PI 16:0_18:2;O2 appears as two chromatographically resolved isomers (3.71 and 3.86 min) that are light-enriched. Alternatively, PI 16:0_18:2;O has the opposite trend, being dark-enriched across all genotypes. The trienoate PI 34:3 is dark-enriched, paralleling the trend observed for one of the oxidized forms PI 16:0_18:3;O. These patterns illustrate the strong molecular-species dependence of PI remodelling and oxidation state under diurnal conditions. Image, application 5

Supplemental Figure S6. Diurnal prenol lipid abundance in WT and SLP1 mutant *Arabidopsis* rosettes. Summed intensities for all annotated prenol lipids in SLP1 wild-type (WT), knockout (KO, *slp1-/-*), and over-expression lines (OE) under light and dark conditions (**p* < 0.05 for Student’s *t*-test). Bars represent mean ± one standard deviation. Significant changes at the lipid species level are displayed in Supplemental Table 1. Image, application 6

Supplemental Figure S7. The acyl tail composition of lipids varies with light and dark conditions. Clustered heatmaps display the distribution of lipids based on top: even- vs. odd-carbon acyl chain length, and bottom: degree of saturation. Features were selected using Random Forest classification, and values were normalized, auto-scaled, and clustered to reveal condition-dependent patterns. Image, application 7

Supplemental Figure S8. Diurnal fluctuations in acyl chain length distribution within select glycerolipid subclasses in *Arabidopsis* rosettes. Plots show the percentage contribution of each acyl chain length category to the total intensity within each lipid class. Only high-confidence annotated lipids were included. Data are shown for wild-type (WT), SLP1 knockout (*slp1-/-*, KO), and SLP1 over-expression (OE) lines under light and dark conditions. Image, application 8

Supplemental Figure S9. Diurnal variation in phospholipid abundance in WT and SLP1 mutant *Arabidopsis* rosettes. Summed intensity of all annotated phospholipids are shown for wild-type (WT), SLP1 knockout (*slp1-/-*, KO), and SLP1 over-expression (OE) lines under light and dark conditions. Bars represent mean ± one standard deviation. One-way ANOVA followed by Tukey’s Honestly Significant Difference test (*p* < 0.05) was used to assess differences among groups. Groups sharing the same letter are not significantly different. Image, application 9

Supplemental Figure S10. Triacylglycerols (TG) are enriched in the *slp1-/-* lipidome under light conditions. Lipid set enrichment analysis (LSEA) comparing wild-type (WT) and *slp1-/-* samples under light conditions identified TG species with long, even-numbered, polyunsaturated acyl chains as enriched in the *slp1-/-* lipidome (FDR-adjusted *p* ≈ 0.07). Image, application 10

Supplemental Figure S11. Galactolipids (MGDG and DGDG) show increased abundance 6 h into the light period in a 16/8 light–dark cycle. Data are shown for wild-type (WT), SLP1 knockout (*slp1-/-*, KO), and over-expression (OE) lines harvested under light and dark conditions. Image, application 11

Supplemental Table S1. Annotated prenol lipid species and their intensities (± standard deviation) found in the *Arabidopsis* rosette tissue using untargeted lipidomics. Data are shown for wild-type (WT), SLP1 knockout (*slp1-/-*, KO), and over-expression (OE) lines harvested under light (L) and dark (D) conditions. Lipids are sorted by likelihood of *bona fide* identification in Arabidopsis. Peak numbers correspond to annotations listed in Supplemental Data 1. The right-hand side of the table shows the results of Student’s *t*-tests comparing light vs. dark (left three columns) and WT vs. SLP1 mutants (right four columns). *p* < 0.05; **p* < 0.01. Image, application 12

Supplementary File S1. List of detected features and raw peak intensities. Annotations are reported without retention time filtering. A) Raw peak intensities for annotated and unknown features. B) Annotated lipids and class distributions for wide-type plants. C) Most abundant lipids in wild-type plants, based on raw (non-normalized) peak intensities. D) List of lipid class abbreviations. Image, application 13

Supplementary File S2. List of annotated species and unknown features with their normalized peak intensities. Image, application 14

Supplementary File S3. Lipids enriched 0.67>FC>1.5 (and set statistical cutoffs) in the light and dark compared across WT and SLP1 mutant lines. Lipids exclusive to each genotype or in common with other genotypes were tabulated and then quantified to construct the Venn diagram in Figure 3. Image, application 15

Supplementary File S4. List of significantly altered lipids for volcano plot analysis. Image, application 16
