# Supplementary material for: Lipidomic profiling of Arabidopsis chloroplast protein phosphatase SLP1 mutants reveals altered diurnal lipid remodeling
Source: BBA Adv. 2026 Jan 9;9:100180. doi: 10.1016/j.bbadva.2026.100180 (PMC12834941; doi:10.1016/j.bbadva.2026.100180)
Supplement: Supplementary file 1 — Supplemental Figure S1. Lipid class distribution of annotated peaks from untargeted lipidomics of Arabidopsis rosette tissue. High confidence lipids (117) were annotated based on MS/MS spectral matches with a score ≥ 400 and m/z error ≤ 2.5 ppm or 3.0 mDa). Low confidence annotations (301) were assigned based on mass matches within m/z error of 2.5 ppm. All annotations passed retention time, adduct, ionization patterns, and biological context filtering. Abbreviations: FA- fatty acids, GL- glycerolipids, GP- glycerophospholipids, SP- sphingolipids, ST- sterols. Additional abbreviations for lipid classes and subclasses are listed in Supplemental Data 1. [file mmc1.pdf]

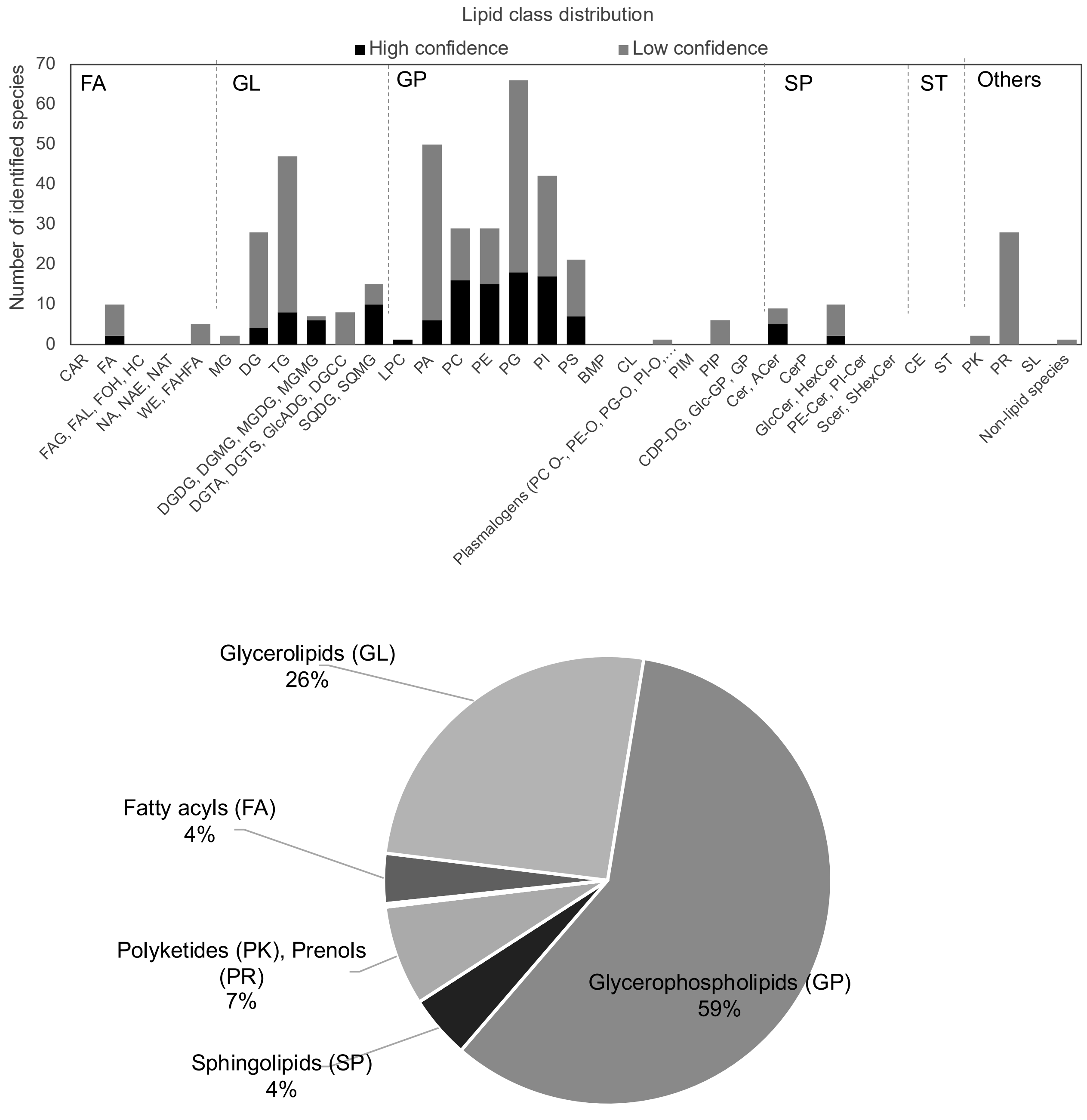

Supplemental Figure S1. Lipid class distribution of annotated peaks from untargeted lipidomics of *Arabidopsis* rosette tissue. High confidence lipids (117) were annotated based on MS/MS spectral matches with a score ≥ 400 and m/z error ≤ 2.5 ppm or 3.0 mDa). Low confidence annotations (301) were assigned based on mass matches within m/z error of 2.5 ppm. All annotations passed retention time, adduct, ionization patterns, and biological context filtering. Abbreviations: FA- fatty acids, GL- glycerolipids, GP- glycerophospholipids, SP- sphingolipids, ST- sterols. Additional abbreviations for lipid classes and subclasses are listed in Supplemental Data 1.
