# Supplementary material for: Lipidomic profiling of Arabidopsis chloroplast protein phosphatase SLP1 mutants reveals altered diurnal lipid remodeling
Source: BBA Adv. 2026 Jan 9;9:100180. doi: 10.1016/j.bbadva.2026.100180 (PMC12834941; doi:10.1016/j.bbadva.2026.100180)
Supplement: Supplementary file 2 — Supplemental Figure S2. Annotated lipids with the 30 top raw intensities in wild-type plants during light and dark cycles. * p (Light/Dark) <0.05, ** p (Light/Dark) <0.05 and fold-change (Light/Dark) ≤0.667 or ≥1.50. [file mmc2.pdf]

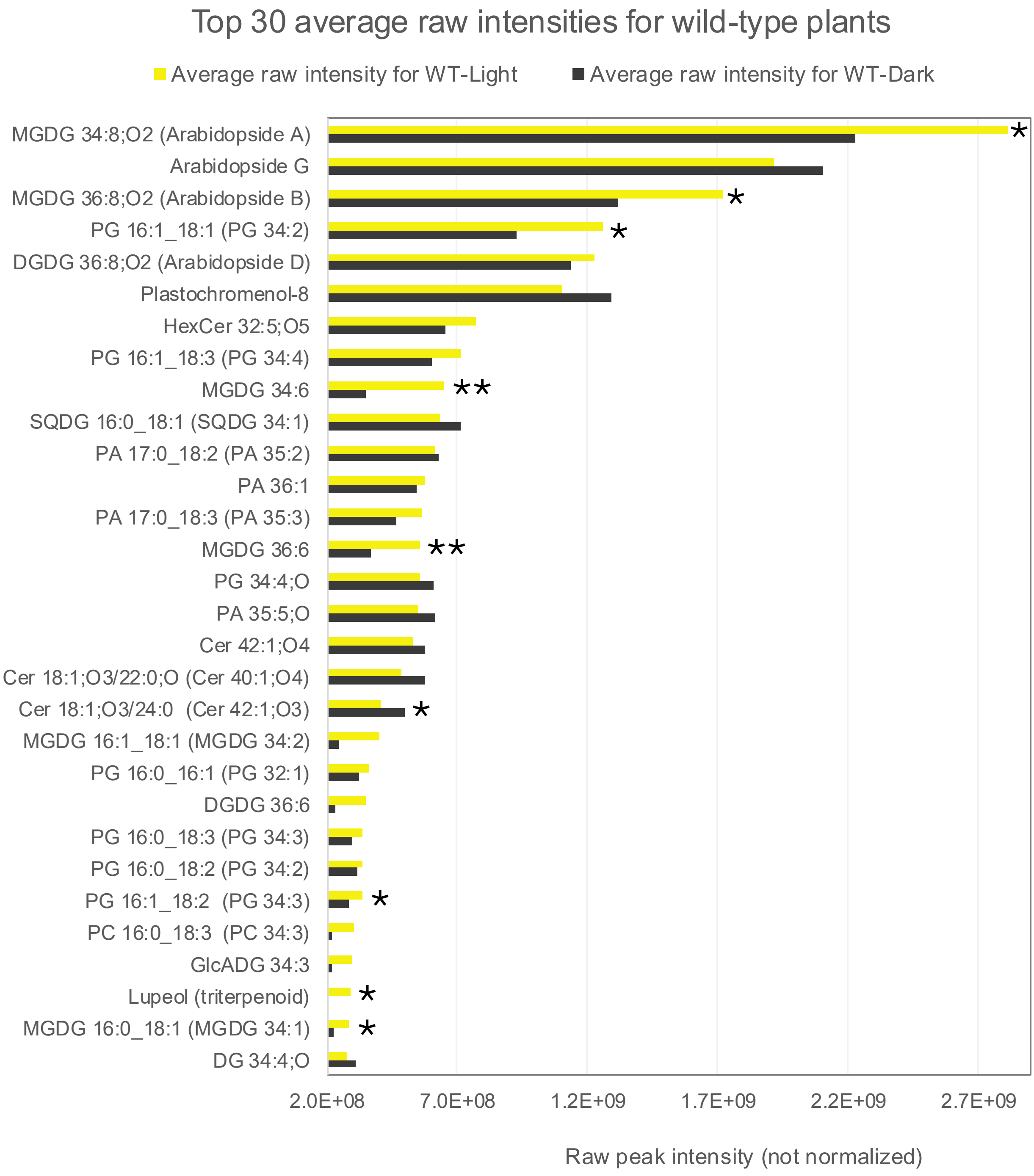

Supplemental Figure S2. Annotated lipids with the 30 top raw intensities in wild-type plants during light and dark cycles. * p (Light/Dark) <0.05, ** p (Light/Dark) <0.05 and fold-change (Light/Dark) ≤0.667 or ≥1.50.
