# Supplementary material for: Lipidomic profiling of Arabidopsis chloroplast protein phosphatase SLP1 mutants reveals altered diurnal lipid remodeling
Source: BBA Adv. 2026 Jan 9;9:100180. doi: 10.1016/j.bbadva.2026.100180 (PMC12834941; doi:10.1016/j.bbadva.2026.100180)
Supplement: Supplementary file 3 — Supplemental Figure S3. SLP1 influences the differential diurnal enrichment of lipids in Arabidopsis rosettes. A) Volcano plot analysis reveals significantly altered lipids for light and dark conditions (fold-change ≥1.5 or ≤0.67, raw p < 0.05, FDR-p < 0.10) within each Arabidopsis genotype. A complete list of lipid annotations, fold-changes, and p-values is provided in Supplemental Data 2. Selected lipids of particular interest are labelled as: a- PIP 38:2;O, b-PI 32:1;O, c-DG 32:0. B) Lipid set enrichment analysis (LSEA) indicates that long-chained, even carbon-numbered, polyunsaturated phosphatidylglycerol species are specifically enriched in slp1-/- dark rosettes. LSEA was performed using a custom Python script that applies a modified gene set enrichment logic to lipidomics data. Lipid species were grouped into sets based on shared structural features (e.g., headgroup, chain length, saturation, or subclass). Enrichment was assessed using a one-sided Fisher’s exact test comparing significantly altered lipids (fold change ≥1.5, p < 0.05, FDR < 0.10) against the background of all detected lipids. Lipid sets with FDR-adjusted p < 0.10 were considered significantly enriched. [file mmc3.pdf]

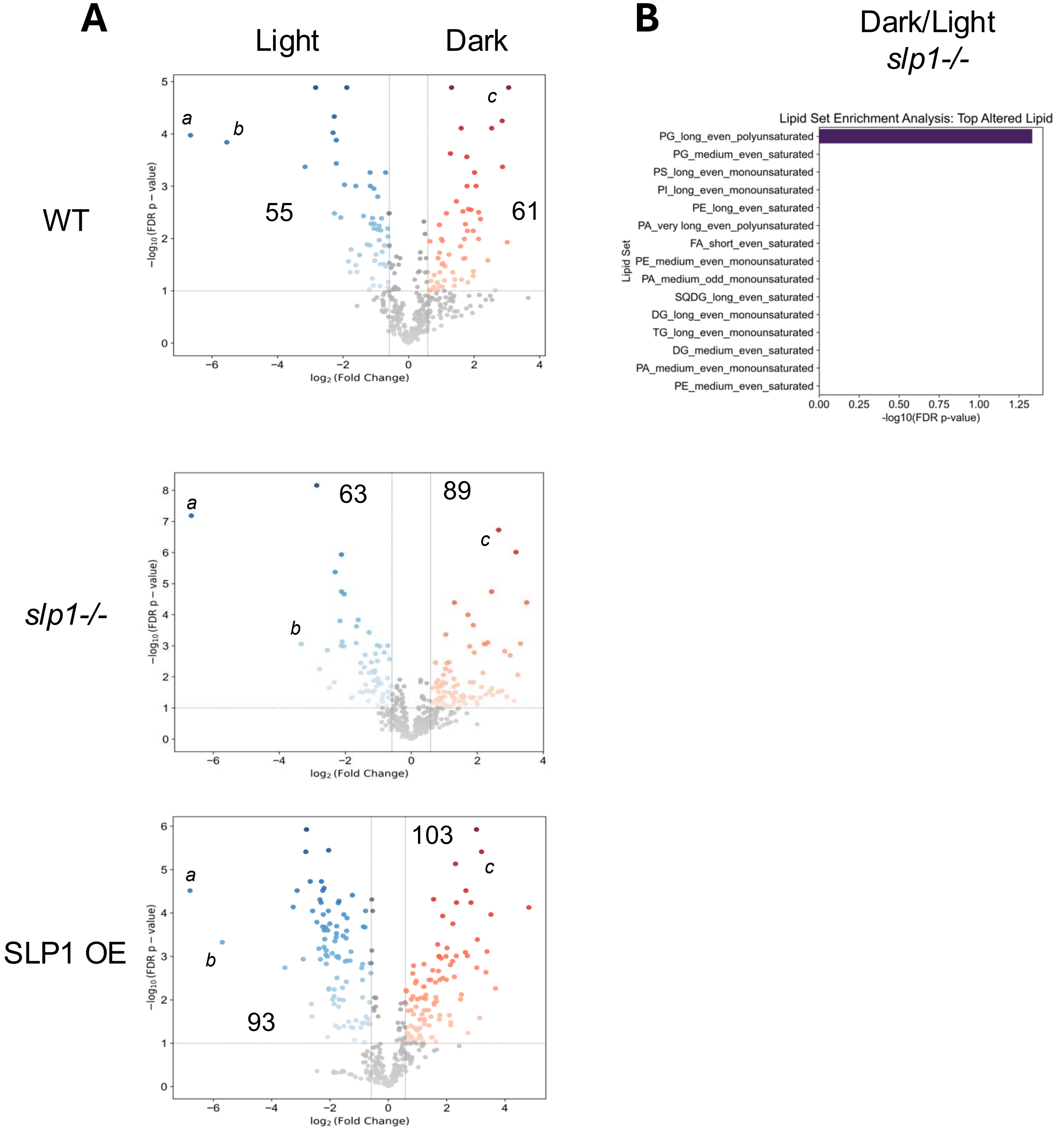

Supplemental Figure S3. SLP1 influences the differential diurnal enrichment of lipids in *Arabidopsis* rosettes. A) Volcano plot analysis reveals significantly altered lipids for light and dark conditions (fold-change ≥1.5 or ≤0.67, raw p <0.05, FDR-p <0.10) within each *Arabidopsis* genotype. A complete list of lipid annotations, fold-changes, and p-values is provided in Supplemental Data 2. Selected lipids of particular interest are labelled as: a- PIP 38:2;O, b-PI 32:1;O, c-DG 32:0. B) Lipid set enrichment analysis (LSEA) indicates that long-chained, even carbon-numbered, polyunsaturated phosphatidylglycerol species are specifically enriched in *slp1-/-* dark rosettes. LSEA was performed using a custom Python script that applies a modified gene set enrichment logic to lipidomics data. Lipid species were grouped into sets based on shared structural features (e.g., headgroup, chain length, saturation, or subclass). Enrichment was assessed using a one-sided Fisher's exact test comparing significantly altered lipids (fold change ≥1.5, p < 0.05, FDR < 0.10) against the background of all detected lipids. Lipid sets with FDR-adjusted p < 0.10 were considered significantly enriched.
