# Supplementary material for: Lipidomic profiling of Arabidopsis chloroplast protein phosphatase SLP1 mutants reveals altered diurnal lipid remodeling
Source: BBA Adv. 2026 Jan 9;9:100180. doi: 10.1016/j.bbadva.2026.100180 (PMC12834941; doi:10.1016/j.bbadva.2026.100180)
Supplement: Supplementary file 4 — Supplemental Figure S4. Phosphatidylinositol phosphates (PIP) show diurnal abundance and saturation changes in Arabidopsis rosettes. A) Violin plot depicting the variation in summed intensity of PIPs under light and dark conditions for all three genotypes. B) Proportion of PIP peak intensities with (un)saturated carbon tails across light and dark conditions in SLP1 wild-type (WT), knockout (KO, slp1-/-), and over-expression lines (OE). Supplemental Data 1 lists all PIP lipid annotations used for analyses. C) [file mmc4.pdf]

A

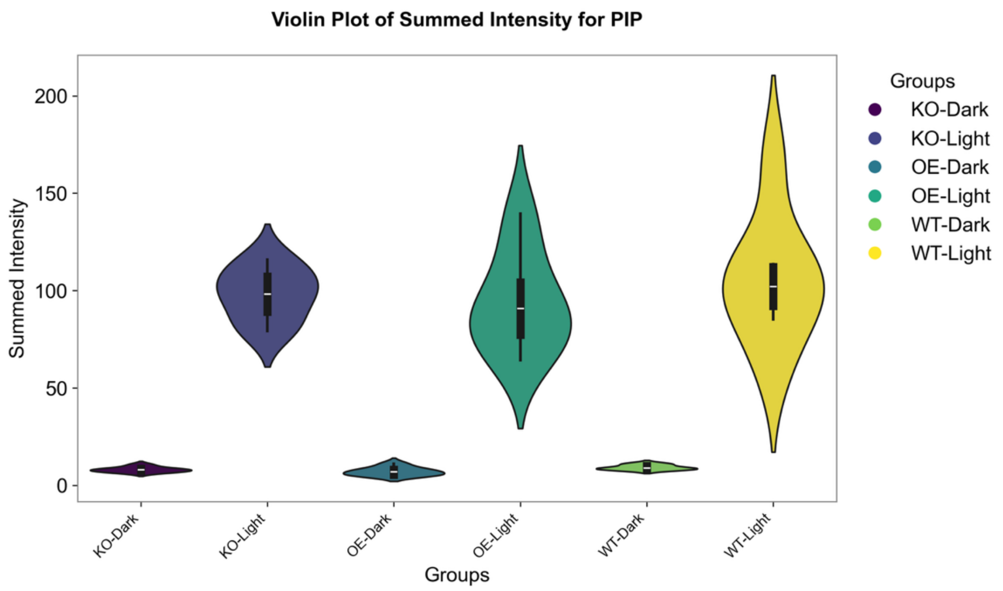

B

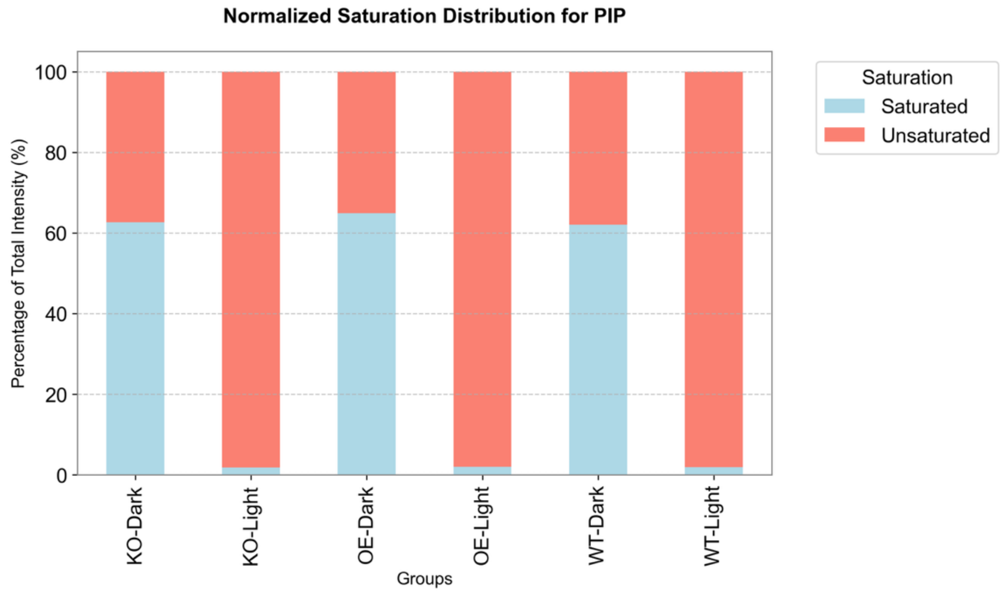

Supplemental Figure S4. Phosphatidylinositol phosphates (PIP) show diurnal abundance and saturation changes in *Arabidopsis* rosettes. A) Violin plot depicting the variation in summed intensity of PIPs under light and dark conditions for all three genotypes. B) Proportion of PIP peak intensities with (un)saturated carbon tails across light and dark conditions in SLP1 wild-type (WT), knockout (KO, *slp1-/-*), and over-expression lines (OE). Supplemental Data 1 lists all PIP lipid annotations used for analyses. C)
