# Supplementary material for: Lipidomic profiling of Arabidopsis chloroplast protein phosphatase SLP1 mutants reveals altered diurnal lipid remodeling
Source: BBA Adv. 2026 Jan 9;9:100180. doi: 10.1016/j.bbadva.2026.100180 (PMC12834941; doi:10.1016/j.bbadva.2026.100180)
Supplement: Supplementary file 5 — Supplemental Figure S5. Total abundances of oxidized lipids in Arabidopsis rosettes fluctuate diurnally. A) Summed intensities for oxidized glycerolipids, glycerophospholipids, and fatty acyls for light and dark in WT and SLP1 mutant lines. The bars represent mean values ± one standard deviation. B) The phosphatidylglycerol PG 16:0_18:2;O2 represents a light-enriched oxylipid. C) Unsaturated phosphatidylinositols (PI) with 34 carbons exhibit distinct light–dark behaviours. PI 34:2 is strongly enriched in the light across all genotypes. Likewise, the oxidized species PI 16:0_18:2;O2 appears as two chromatographically resolved isomers (3.71 and 3.86 min) that are light-enriched. Alternatively, PI 16:0_18:2;O has the opposite trend, being dark-enriched across all genotypes. The trienoate PI 34:3 is dark-enriched, paralleling the trend observed for one of the oxidized forms PI 16:0_18:3;O. These patterns illustrate the strong molecular-species dependence of PI remodelling and oxidation state under diurnal conditions. [file mmc5.pdf]

A

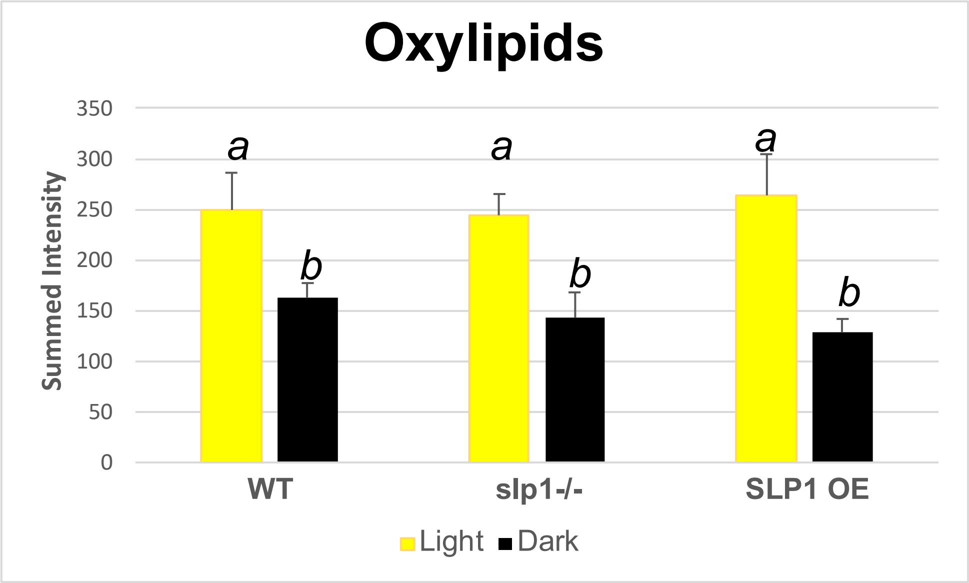
Oxylipids
Summed Intensity
350
300
250
200
150
100
50
0
a
b
a
b
a
b
WT
slp1-/-
SLP1 OE
Light
Dark

B

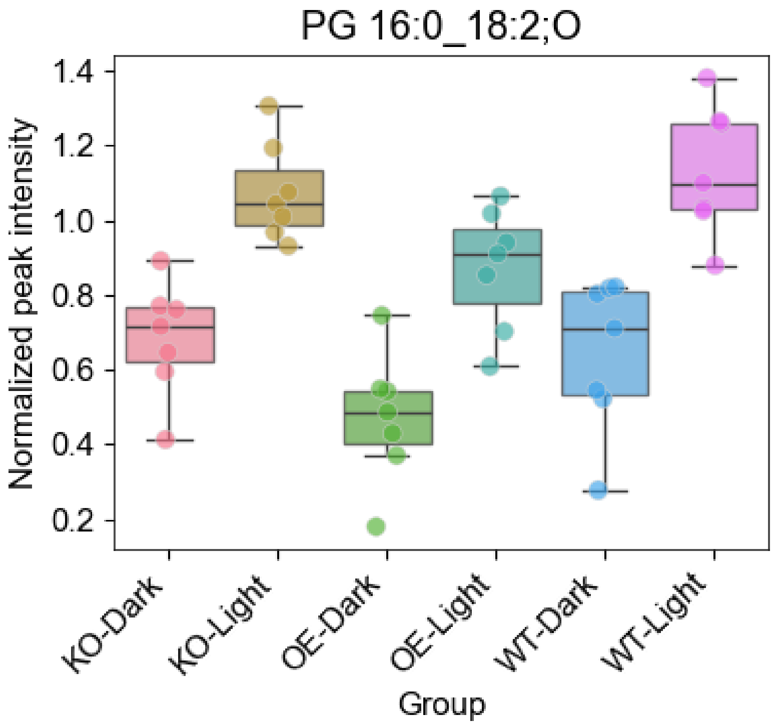
PG 16:0_18:2;O
Normalized peak intensity
1.4
1.2
1.0
0.8
0.6
0.4
0.2
KO-Dark
KO-Light
OE-Dark
OE-Light
WT-Dark
WT-Light
Group

C

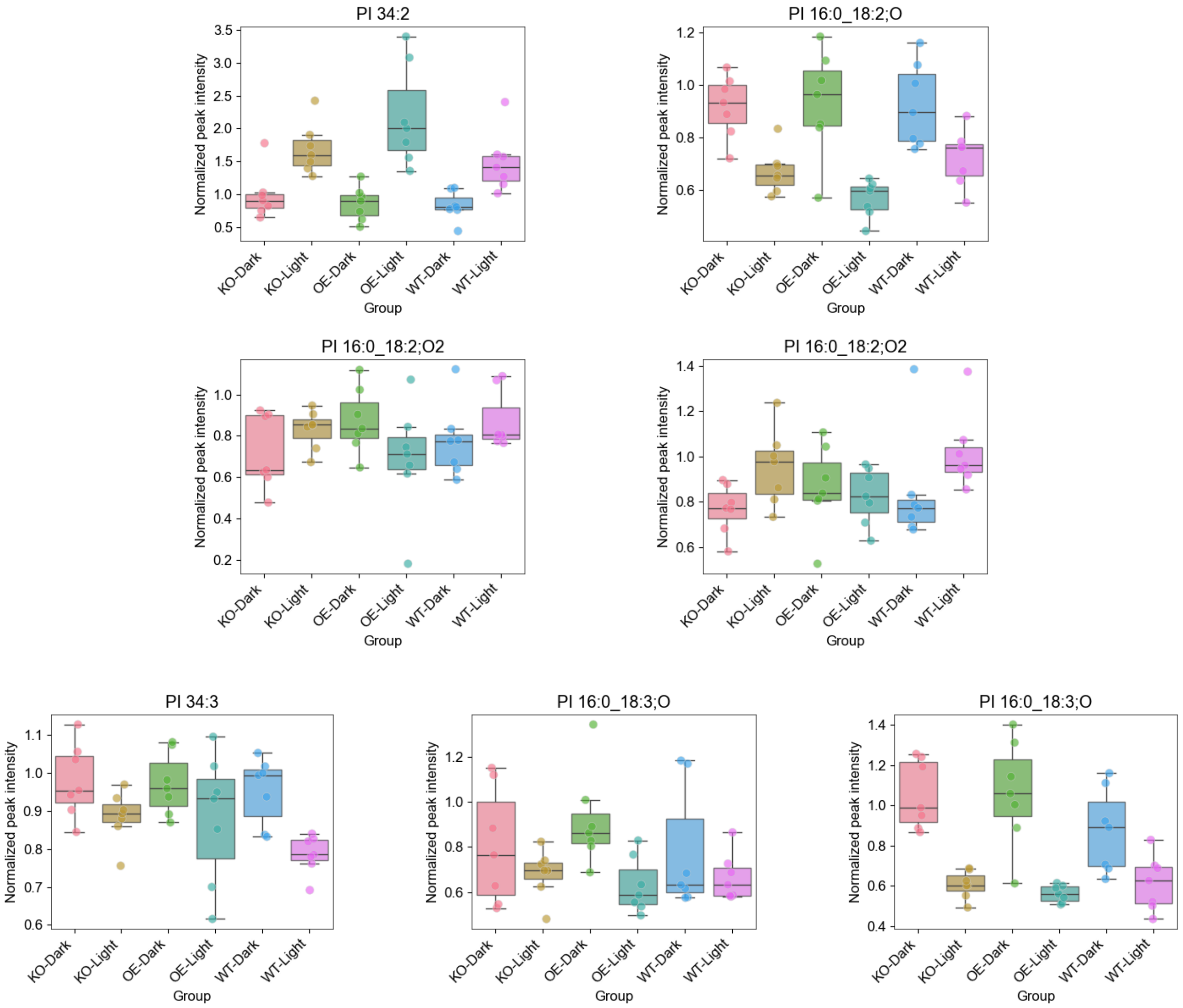

Supplemental Figure S5. Total abundances of oxidized lipids in Arabidopsis rosettes fluctuate diurnally. A) Summed intensities for oxidized glycerolipids, glycerophospholipids, and fatty acyls for light and dark in WT and SLP1 mutant lines. The bars represent mean values ± one standard deviation. B) The phosphatidylglycerol PG 16:0_18:2;O2 represents a light-enriched oxylipid. C) Unsaturated phosphatidylinositols (PI) with 34 carbons exhibit distinct light–dark behaviours. PI 34:2 is strongly enriched in the light across all genotypes. Likewise, the oxidized species PI 16:0_18:2;O2 appears as two chromatographically resolved isomers (3.71 and 3.86 min) that are light-enriched. Alternatively, PI 16:0_18:2;O has the opposite trend, being dark-enriched across all genotypes. The trienoate PI 34:3 is dark-enriched, paralleling the trend observed for one of the oxidized forms PI 16:0_18:3;O. These patterns illustrate the strong molecular-species dependence of PI remodelling and oxidation state under diurnal conditions.
