# Supplementary material for: Lipidomic profiling of Arabidopsis chloroplast protein phosphatase SLP1 mutants reveals altered diurnal lipid remodeling
Source: BBA Adv. 2026 Jan 9;9:100180. doi: 10.1016/j.bbadva.2026.100180 (PMC12834941; doi:10.1016/j.bbadva.2026.100180)
Supplement: Supplementary file 6 — Supplemental Figure S6. Diurnal prenol lipid abundance in WT and SLP1 mutant Arabidopsis rosettes. Summed intensities for all annotated prenol lipids in SLP1 wild-type (WT), knockout (KO, slp1-/-), and over-expression lines (OE) under light and dark conditions (*p < 0.05 for Student’s t-test). Bars represent mean ± one standard deviation. Significant changes at the lipid species level are displayed in Supplemental Table 1. [file mmc6.pdf]

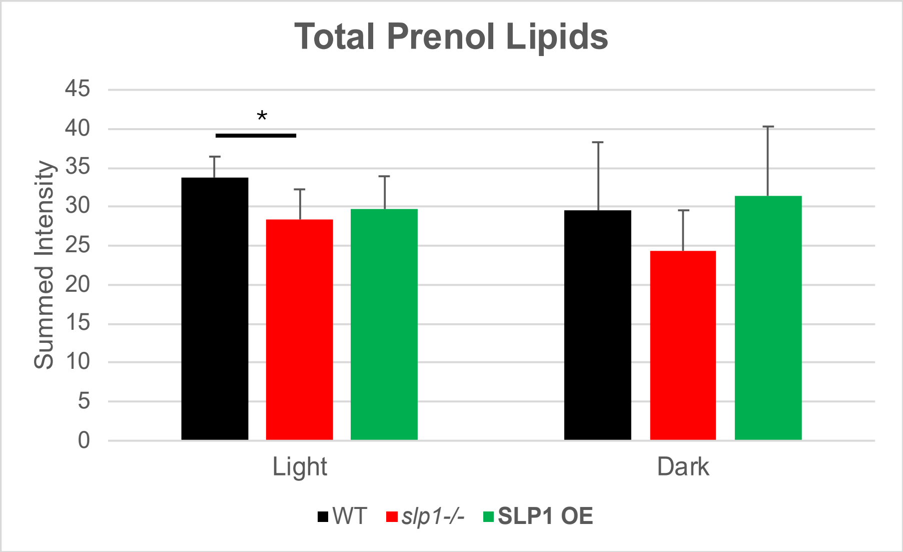

Supplemental Figure S6. Diurnal prenol lipid abundance in WT and SLP1 mutant *Arabidopsis* rosettes. Summed intensities for all annotated prenol lipids in SLP1 wild-type (WT), knockout (KO, *slp1-/-*), and over-expression lines (OE) under light and dark conditions (*$p<0.05$ for Student's t-test). Bars represent mean ± one standard deviation. Significant changes at the lipid species level are displayed in Supplemental Table 1.
