# Supplementary material for: Lipidomic profiling of Arabidopsis chloroplast protein phosphatase SLP1 mutants reveals altered diurnal lipid remodeling
Source: BBA Adv. 2026 Jan 9;9:100180. doi: 10.1016/j.bbadva.2026.100180 (PMC12834941; doi:10.1016/j.bbadva.2026.100180)
Supplement: Supplementary file 7 — Supplemental Figure S7. The acyl tail composition of lipids varies with light and dark conditions. Clustered heatmaps display the distribution of lipids based on top: even- vs. odd-carbon acyl chain length, and bottom: degree of saturation. Features were selected using Random Forest classification, and values were normalized, auto-scaled, and clustered to reveal condition-dependent patterns. [file mmc7.pdf]

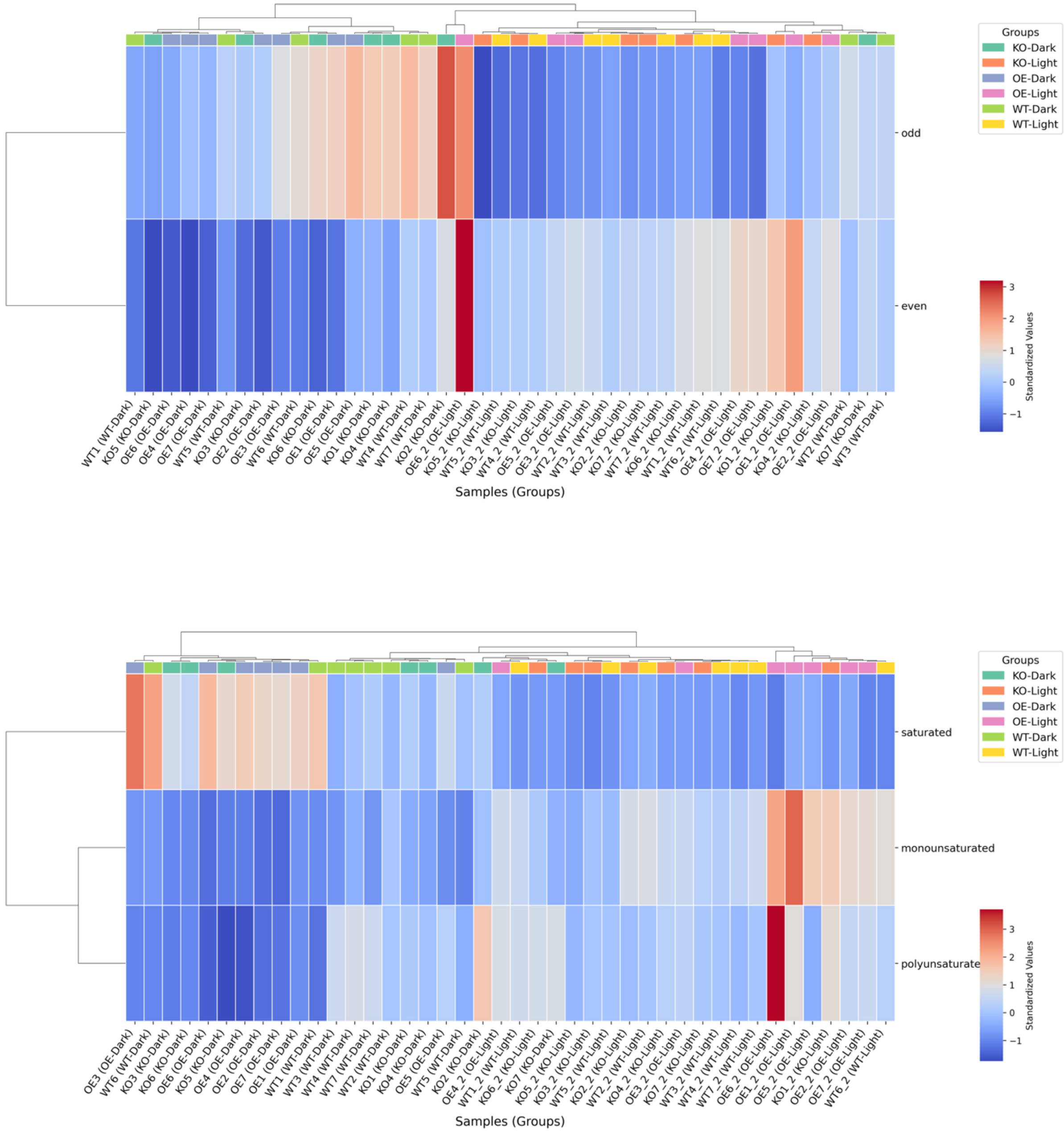

Supplemental Figure S7. The acyl tail composition of lipids varies with light and dark conditions. Clustered heatmaps display the distribution of lipids based on top: even- vs. odd-carbon acyl chain length, and bottom: degree of saturation. Features were selected using Random Forest classification, and values were normalized, auto-scaled, and clustered to reveal condition-dependent patterns.
