# Supplementary material for: Lipidomic profiling of Arabidopsis chloroplast protein phosphatase SLP1 mutants reveals altered diurnal lipid remodeling
Source: BBA Adv. 2026 Jan 9;9:100180. doi: 10.1016/j.bbadva.2026.100180 (PMC12834941; doi:10.1016/j.bbadva.2026.100180)
Supplement: Supplementary file 8 — Supplemental Figure S8. Diurnal fluctuations in acyl chain length distribution within select glycerolipid subclasses in Arabidopsis rosettes. Plots show the percentage contribution of each acyl chain length category to the total intensity within each lipid class. Only high-confidence annotated lipids were included. Data are shown for wild-type (WT), SLP1 knockout (slp1-/-, KO), and SLP1 over-expression (OE) lines under light and dark conditions. [file mmc8.pdf]

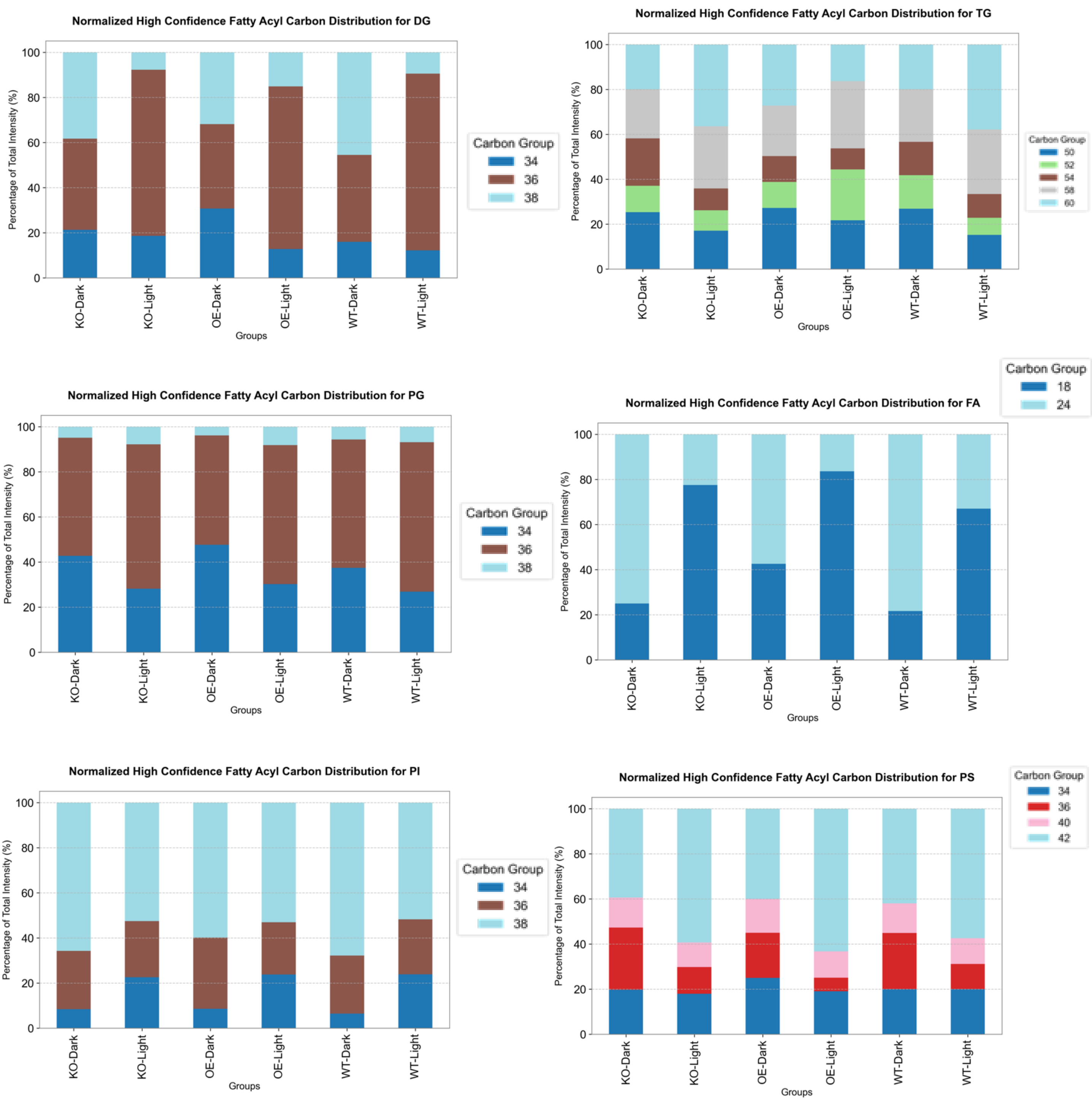


**Supplemental Figure S8. Diurnal fluctuations in acyl chain length distribution within select glycerolipid subclasses in *Arabidopsis* rosettes.** Plots show the percentage contribution of each acyl chain length category to the total intensity within each lipid class. Only high-confidence annotated lipids were included. Data are shown for wild-type (WT), SLP1 knockout (*slp1*-/-, KO), and SLP1 over-expression (OE) lines under light and dark conditions.
