# Supplementary material for: Lipidomic profiling of Arabidopsis chloroplast protein phosphatase SLP1 mutants reveals altered diurnal lipid remodeling
Source: BBA Adv. 2026 Jan 9;9:100180. doi: 10.1016/j.bbadva.2026.100180 (PMC12834941; doi:10.1016/j.bbadva.2026.100180)
Supplement: Supplementary file 9 — Supplemental Figure S9. Diurnal variation in phospholipid abundance in WT and SLP1 mutant Arabidopsis rosettes. Summed intensity of all annotated phospholipids are shown for wild-type (WT), SLP1 knockout (slp1-/-, KO), and SLP1 over-expression (OE) lines under light and dark conditions. Bars represent mean ± one standard deviation. One-way ANOVA followed by Tukey’s Honestly Significant Difference test (p < 0.05) was used to assess differences among groups. Groups sharing the same letter are not significantly different. [file mmc9.pdf]

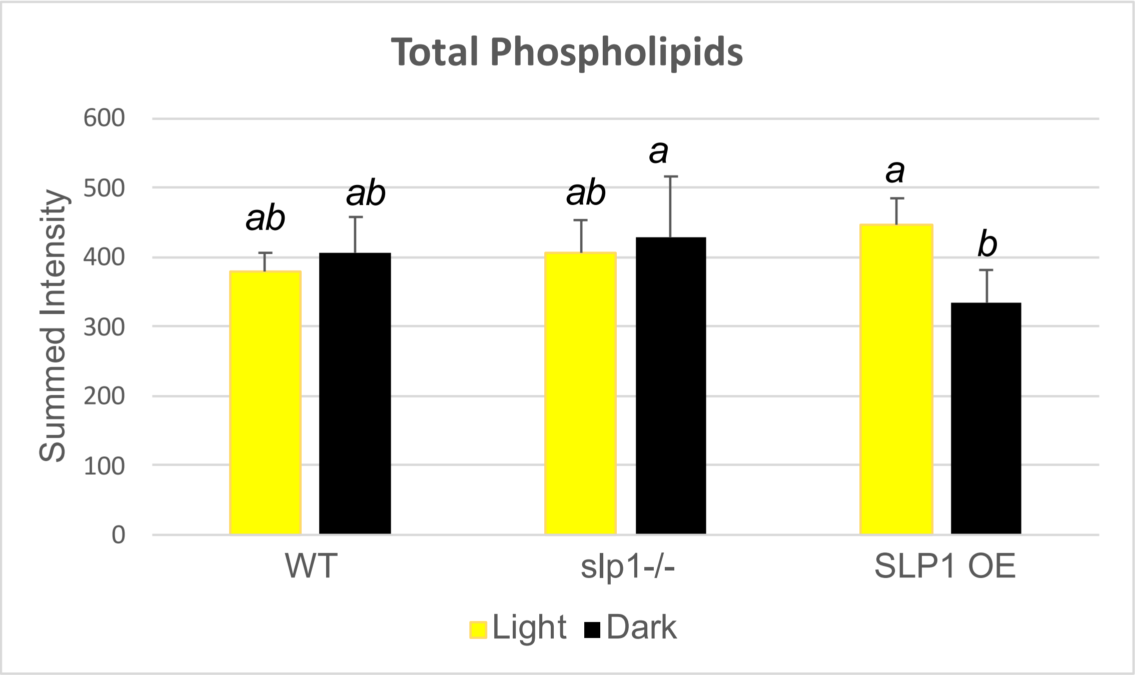

Supplemental Figure S9. Diurnal variation in phospholipid abundance in WT and SLP1 mutant *Arabidopsis* rosettes. Summed intensity of all annotated phospholipids are shown for wild-type (WT), SLP1 knockout (*slp1-/-*, KO), and SLP1 over-expression (OE) lines under light and dark conditions. Bars represent mean ± one standard deviation. One-way ANOVA followed by Tukey's Honestly Significant Difference test ($p < 0.05$) was used to assess differences among groups. Groups sharing the same letter are not significantly different.
