# Supplementary material for: Lipidomic profiling of Arabidopsis chloroplast protein phosphatase SLP1 mutants reveals altered diurnal lipid remodeling
Source: BBA Adv. 2026 Jan 9;9:100180. doi: 10.1016/j.bbadva.2026.100180 (PMC12834941; doi:10.1016/j.bbadva.2026.100180)
Supplement: Supplementary file 10 — Supplemental Figure S10. Triacylglycerols (TG) are enriched in the slp1-/- lipidome under light conditions. Lipid set enrichment analysis (LSEA) comparing wild-type (WT) and slp1-/- samples under light conditions identified TG species with long, even-numbered, polyunsaturated acyl chains as enriched in the slp1-/- lipidome (FDR-adjusted p ≈ 0.07). [file mmc10.pdf]

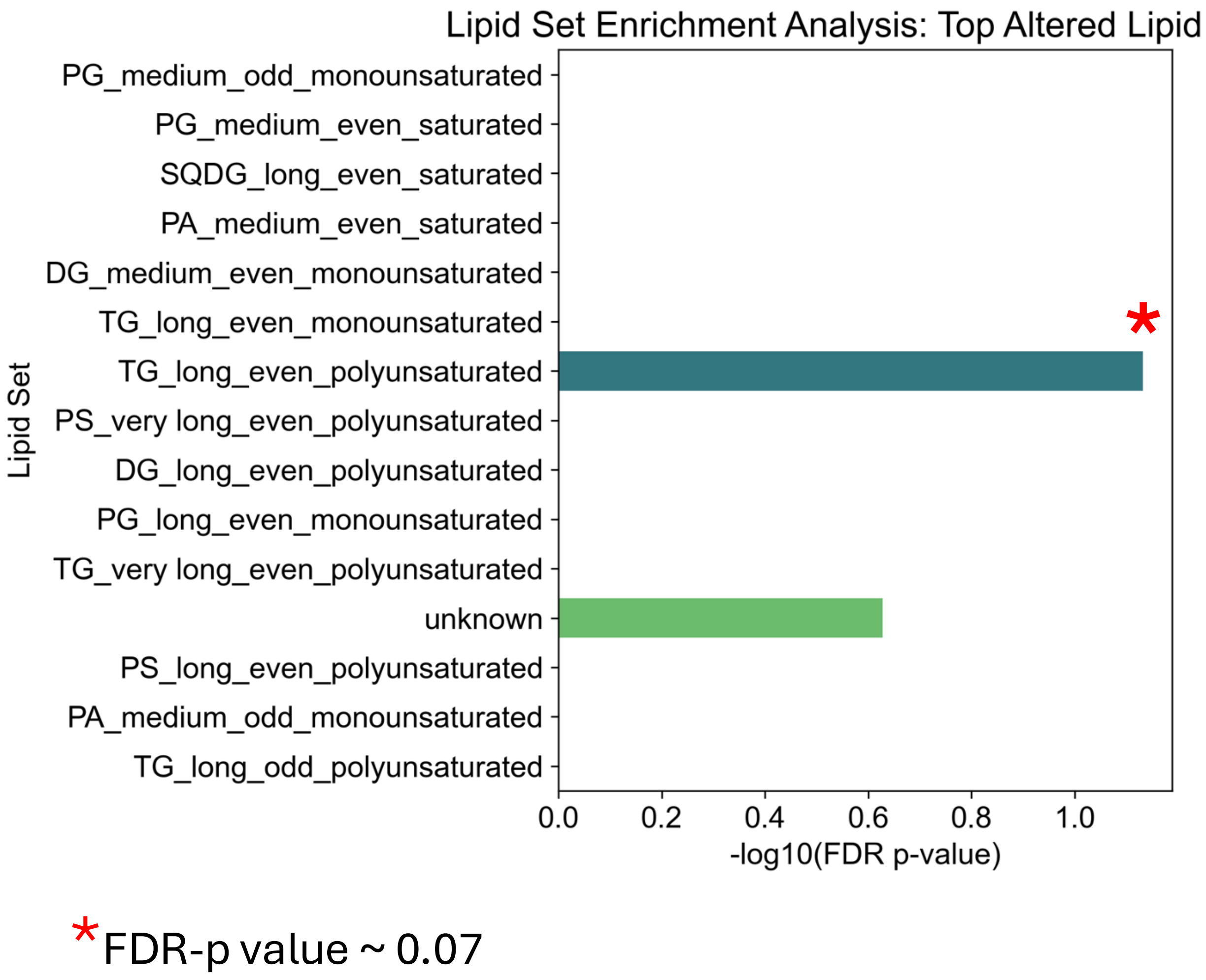

Supplemental Figure S10. Triacylglycerols (TG) are enriched in the *slp1-/-* lipidome under light conditions. Lipid set enrichment analysis (LSEA) comparing wild-type (WT) and *slp1-/-*samples under light conditions identified TG species with long, even-numbered, polyunsaturated acyl chains as enriched in the *slp1-/-* lipidome (FDR-adjusted p ≈ 0.07).
