# Supplementary material for: Lipidomic profiling of Arabidopsis chloroplast protein phosphatase SLP1 mutants reveals altered diurnal lipid remodeling
Source: BBA Adv. 2026 Jan 9;9:100180. doi: 10.1016/j.bbadva.2026.100180 (PMC12834941; doi:10.1016/j.bbadva.2026.100180)
Supplement: Supplementary file 11 — Supplemental Figure S11. Galactolipids (MGDG and DGDG) show increased abundance 6 h into the light period in a 16/8 light–dark cycle. Data are shown for wild-type (WT), SLP1 knockout (slp1-/-, KO), and over-expression (OE) lines harvested under light and dark conditions. [file mmc11.pdf]

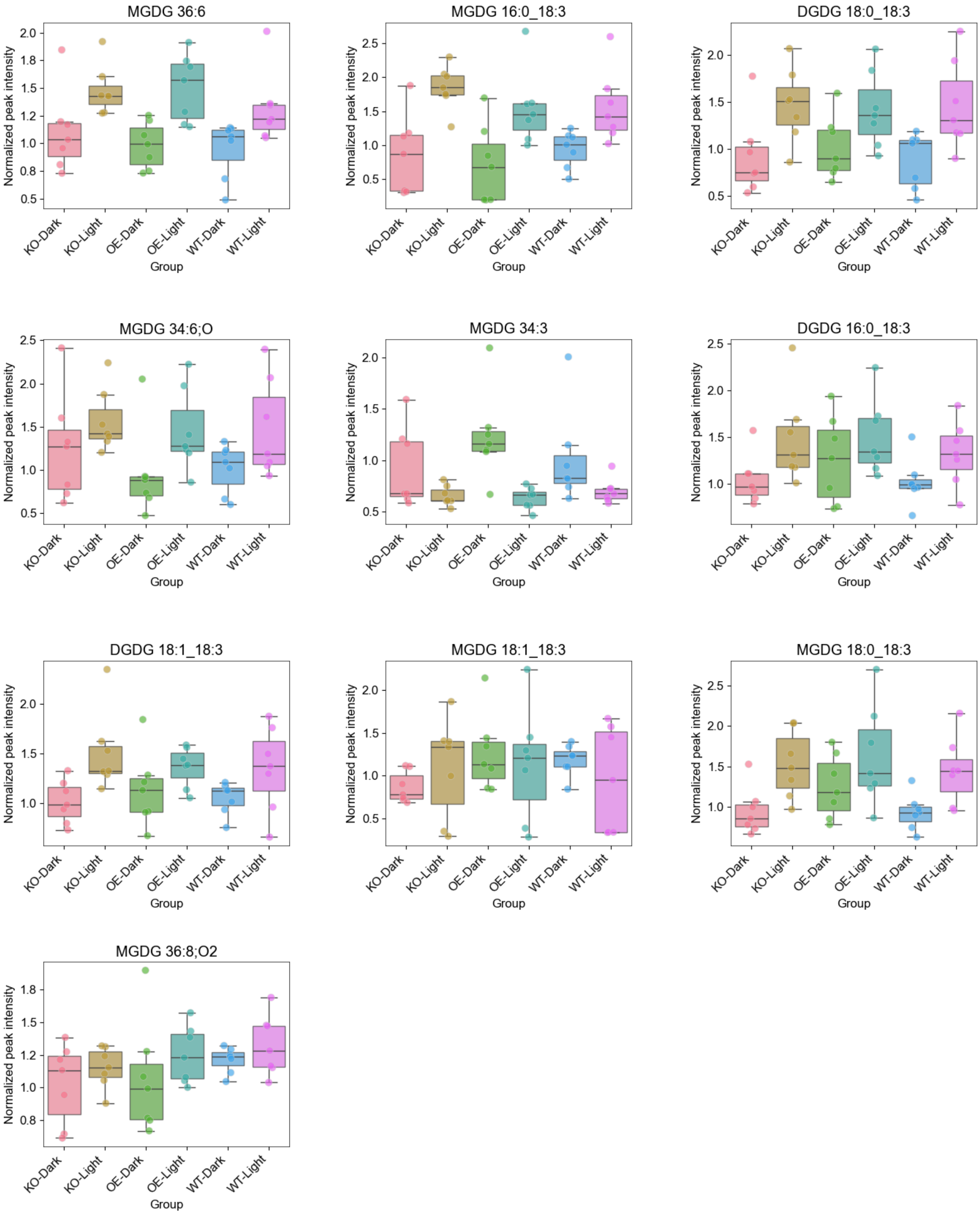

Supplemental Figure S11. Galactolipids (MGDG and DGDG) show increased abundance 6 hours into the light period in a 16/8 light–dark cycle. Data are shown for wild-type (WT), SLP1 knockout (slp1-/- , KO), and over-expression (OE) lines harvested under light (L) and dark (D) conditions.
