# Supplementary material for: Lipidomic profiling of Arabidopsis chloroplast protein phosphatase SLP1 mutants reveals altered diurnal lipid remodeling
Source: BBA Adv. 2026 Jan 9;9:100180. doi: 10.1016/j.bbadva.2026.100180 (PMC12834941; doi:10.1016/j.bbadva.2026.100180)
Supplement: Supplementary file 12 — Supplemental Table S1. Annotated prenol lipid species and their intensities (± standard deviation) found in the Arabidopsis rosette tissue using untargeted lipidomics. Data are shown for wild-type (WT), SLP1 knockout (slp1-/-, KO), and over-expression (OE) lines harvested under light (L) and dark (D) conditions. Lipids are sorted by likelihood of bona fide identification in Arabidopsis. Peak numbers correspond to annotations listed in Supplemental Data 1. The right-hand side of the table shows the results of Student’s t-tests comparing light vs. dark (left three columns) and WT vs. SLP1 mutants (right four columns). p < 0.05; *p < 0.01. [file mmc12.pptx]

## Slide 1
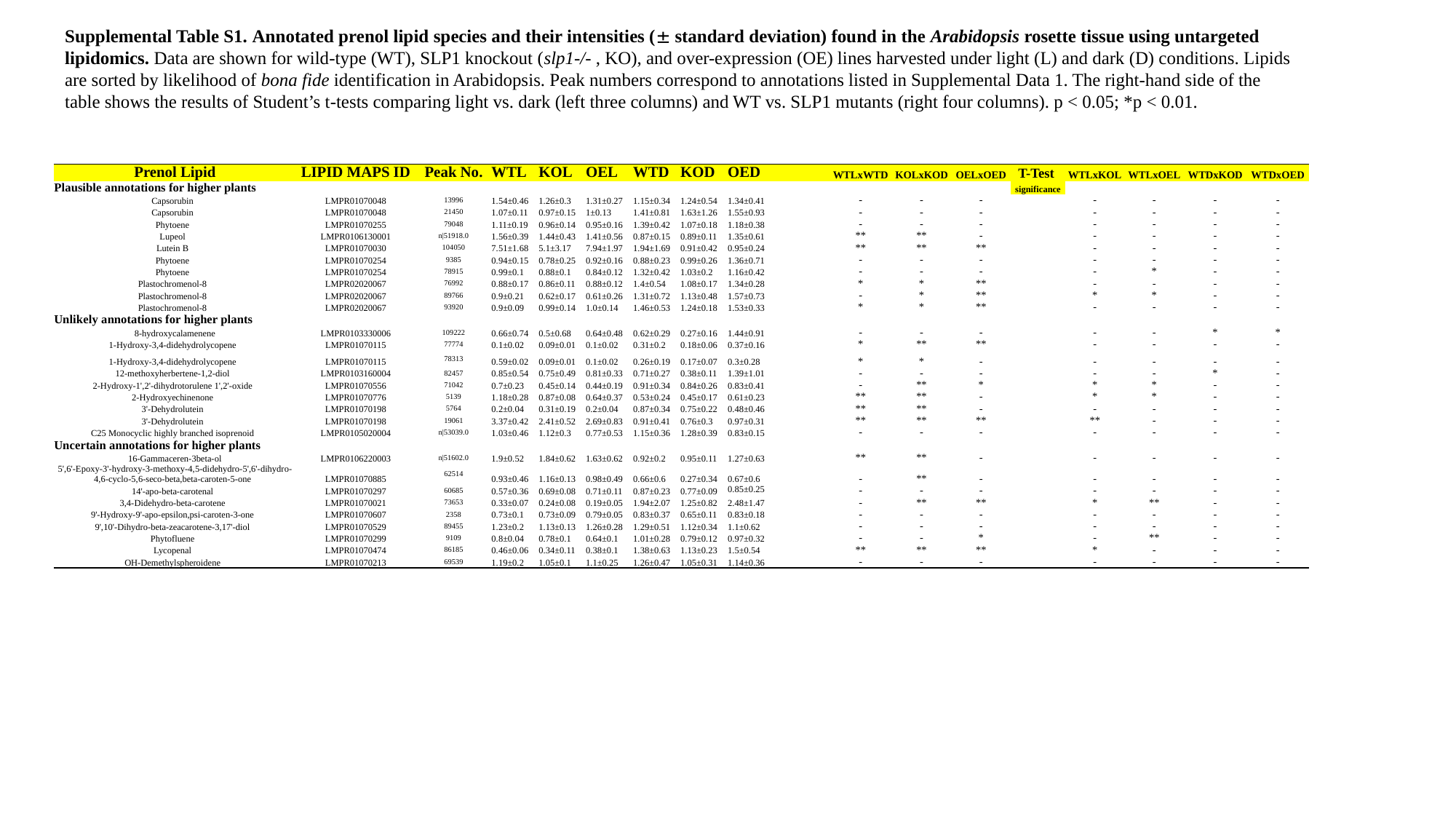

Supplemental Table S1. Annotated prenol lipid species and their intensities (± standard deviation) found in the Arabidopsis rosette tissue using untargeted lipidomics. Data are shown for wild-type (WT), SLP1 knockout (slp1-/- , KO), and over-expression (OE) lines harvested under light (L) and dark (D) conditions. Lipids are sorted by likelihood of bona fide identification in Arabidopsis. Peak numbers correspond to annotations listed in Supplemental Data 1. The right-hand side of the table shows the results of Student’s t-tests comparing light vs. dark (left three columns) and WT vs. SLP1 mutants (right four columns). p < 0.05; *p < 0.01.
| Prenol Lipid | LIPID MAPS ID | Peak No. | WTL | KOL | OEL | WTD | KOD | OED | | WTLxWTD | KOLxKOD | OELxOED | T-Test | WTLxKOL | WTLxOEL | WTDxKOD | WTDxOED |
| --- | --- | --- | --- | --- | --- | --- | --- | --- | --- | --- | --- | --- | --- | --- | --- | --- | --- |
| Plausible annotations for higher plants | | | | | | | | | | | | | significance | | | | |
| Capsorubin | LMPR01070048 | 13996 | 1.54±0.46 | 1.26±0.3 | 1.31±0.27 | 1.15±0.34 | 1.24±0.54 | 1.34±0.41 | | - | - | - | | - | - | - | - |
| Capsorubin | LMPR01070048 | 21450 | 1.07±0.11 | 0.97±0.15 | 1±0.13 | 1.41±0.81 | 1.63±1.26 | 1.55±0.93 | | - | - | - | | - | - | - | - |
| Phytoene | LMPR01070255 | 79048 | 1.11±0.19 | 0.96±0.14 | 0.95±0.16 | 1.39±0.42 | 1.07±0.18 | 1.18±0.38 | | - | - | - | | - | - | - | - |
| Lupeol | LMPR0106130001 | n|51918.0 | 1.56±0.39 | 1.44±0.43 | 1.41±0.56 | 0.87±0.15 | 0.89±0.11 | 1.35±0.61 | | \*\* | \*\* | - | | - | - | - | - |
| Lutein B | LMPR01070030 | 104050 | 7.51±1.68 | 5.1±3.17 | 7.94±1.97 | 1.94±1.69 | 0.91±0.42 | 0.95±0.24 | | \*\* | \*\* | \*\* | | - | - | - | - |
| Phytoene | LMPR01070254 | 9385 | 0.94±0.15 | 0.78±0.25 | 0.92±0.16 | 0.88±0.23 | 0.99±0.26 | 1.36±0.71 | | - | - | - | | - | - | - | - |
| Phytoene | LMPR01070254 | 78915 | 0.99±0.1 | 0.88±0.1 | 0.84±0.12 | 1.32±0.42 | 1.03±0.2 | 1.16±0.42 | | - | - | - | | - | \* | - | - |
| Plastochromenol-8 | LMPR02020067 | 76992 | 0.88±0.17 | 0.86±0.11 | 0.88±0.12 | 1.4±0.54 | 1.08±0.17 | 1.34±0.28 | | \* | \* | \*\* | | - | - | - | - |
| Plastochromenol-8 | LMPR02020067 | 89766 | 0.9±0.21 | 0.62±0.17 | 0.61±0.26 | 1.31±0.72 | 1.13±0.48 | 1.57±0.73 | | - | \* | \*\* | | \* | \* | - | - |
| Plastochromenol-8 | LMPR02020067 | 93920 | 0.9±0.09 | 0.99±0.14 | 1.0±0.14 | 1.46±0.53 | 1.24±0.18 | 1.53±0.33 | | \* | \* | \*\* | | - | - | - | - |
| Unlikely annotations for higher plants | | | | | | | | | | | | | | | | | |
| 8-hydroxycalamenene | LMPR0103330006 | 109222 | 0.66±0.74 | 0.5±0.68 | 0.64±0.48 | 0.62±0.29 | 0.27±0.16 | 1.44±0.91 | | - | - | - | | - | - | \* | \* |
| 1-Hydroxy-3,4-didehydrolycopene | LMPR01070115 | 77774 | 0.1±0.02 | 0.09±0.01 | 0.1±0.02 | 0.31±0.2 | 0.18±0.06 | 0.37±0.16 | | \* | \*\* | \*\* | | - | - | - | - |
| 1-Hydroxy-3,4-didehydrolycopene | LMPR01070115 | 78313 | 0.59±0.02 | 0.09±0.01 | 0.1±0.02 | 0.26±0.19 | 0.17±0.07 | 0.3±0.28 | | \* | \* | - | | - | - | - | - |
| 12-methoxyherbertene-1,2-diol | LMPR0103160004 | 82457 | 0.85±0.54 | 0.75±0.49 | 0.81±0.33 | 0.71±0.27 | 0.38±0.11 | 1.39±1.01 | | - | - | - | | - | - | \* | - |
| 2-Hydroxy-1',2'-dihydrotorulene 1',2'-oxide | LMPR01070556 | 71042 | 0.7±0.23 | 0.45±0.14 | 0.44±0.19 | 0.91±0.34 | 0.84±0.26 | 0.83±0.41 | | - | \*\* | \* | | \* | \* | - | - |
| 2-Hydroxyechinenone | LMPR01070776 | 5139 | 1.18±0.28 | 0.87±0.08 | 0.64±0.37 | 0.53±0.24 | 0.45±0.17 | 0.61±0.23 | | \*\* | \*\* | - | | \* | \* | - | - |
| 3'-Dehydrolutein | LMPR01070198 | 5764 | 0.2±0.04 | 0.31±0.19 | 0.2±0.04 | 0.87±0.34 | 0.75±0.22 | 0.48±0.46 | | \*\* | \*\* | - | | - | - | - | - |
| 3'-Dehydrolutein | LMPR01070198 | 19061 | 3.37±0.42 | 2.41±0.52 | 2.69±0.83 | 0.91±0.41 | 0.76±0.3 | 0.97±0.31 | | \*\* | \*\* | \*\* | | \*\* | - | - | - |
| C25 Monocyclic highly branched isoprenoid | LMPR0105020004 | n|53039.0 | 1.03±0.46 | 1.12±0.3 | 0.77±0.53 | 1.15±0.36 | 1.28±0.39 | 0.83±0.15 | | - | - | - | | - | - | - | - |
| Uncertain annotations for higher plants | | | | | | | | | | | | | | | | | |
| 16-Gammaceren-3beta-ol | LMPR0106220003 | n|51602.0 | 1.9±0.52 | 1.84±0.62 | 1.63±0.62 | 0.92±0.2 | 0.95±0.11 | 1.27±0.63 | | \*\* | \*\* | - | | - | - | - | - |
| 5',6'-Epoxy-3'-hydroxy-3-methoxy-4,5-didehydro-5',6'-dihydro-4,6-cyclo-5,6-seco-beta,beta-caroten-5-one | LMPR01070885 | 62514 | 0.93±0.46 | 1.16±0.13 | 0.98±0.49 | 0.66±0.6 | 0.27±0.34 | 0.67±0.6 | | - | \*\* | - | | - | - | - | - |
| 14'-apo-beta-carotenal | LMPR01070297 | 60685 | 0.57±0.36 | 0.69±0.08 | 0.71±0.11 | 0.87±0.23 | 0.77±0.09 | 0.85±0.25 | | - | - | - | | - | - | - | - |
| 3,4-Didehydro-beta-carotene | LMPR01070021 | 73653 | 0.33±0.07 | 0.24±0.08 | 0.19±0.05 | 1.94±2.07 | 1.25±0.82 | 2.48±1.47 | | - | \*\* | \*\* | | \* | \*\* | - | - |
| 9'-Hydroxy-9'-apo-epsilon,psi-caroten-3-one | LMPR01070607 | 2358 | 0.73±0.1 | 0.73±0.09 | 0.79±0.05 | 0.83±0.37 | 0.65±0.11 | 0.83±0.18 | | - | - | - | | - | - | - | - |
| 9',10'-Dihydro-beta-zeacarotene-3,17'-diol | LMPR01070529 | 89455 | 1.23±0.2 | 1.13±0.13 | 1.26±0.28 | 1.29±0.51 | 1.12±0.34 | 1.1±0.62 | | - | - | - | | - | - | - | - |
| Phytofluene | LMPR01070299 | 9109 | 0.8±0.04 | 0.78±0.1 | 0.64±0.1 | 1.01±0.28 | 0.79±0.12 | 0.97±0.32 | | - | - | \* | | - | \*\* | - | - |
| Lycopenal | LMPR01070474 | 86185 | 0.46±0.06 | 0.34±0.11 | 0.38±0.1 | 1.38±0.63 | 1.13±0.23 | 1.5±0.54 | | \*\* | \*\* | \*\* | | \* | - | - | - |
| OH-Demethylspheroidene | LMPR01070213 | 69539 | 1.19±0.2 | 1.05±0.1 | 1.1±0.25 | 1.26±0.47 | 1.05±0.31 | 1.14±0.36 | | - | - | - | | - | - | - | - |
